# Transcriptomic and Proteomic Changes in the Brain Along with Increasing Phenotypic Severity in a Rat Model of Neonatal Hyperbilirubinemia

**DOI:** 10.3390/ijms26136262

**Published:** 2025-06-28

**Authors:** John Paul Llido, Giorgia Valerio, David Křepelka, Aleš Dvořák, Cristina Bottin, Fabrizio Zanconati, Julia Theresa Regalado, Audrey Franceschi Biagioni, Mohammed Qaisiya, Libor Vítek, Claudio Tiribelli, Silvia Gazzin

**Affiliations:** 1 The Liver Brain Unit “Rita Moretti”, Fondazione Italiana Fegato-Onlus, Bldg. Q, AREA Science Park, 34149 Basovizza, Italy; johnpaul.llido@fegato.it (J.P.L.); giorgia.valerio@fegato.it (G.V.); julia.regalado@fegato.it (J.T.R.); ctliver@fegato.it (C.T.); 2 Department of Science and Technology, Philippine Council for Health Research and Development, Bicutan, Taguig City 1631, Philippines; 3 Department of Life Sciences, University of Trieste, 34139 Trieste, Italy; 4 Institute of Medical Biochemistry and Laboratory Diagnostics, General University Hospital in Prague, 1st Faculty of Medicine, Charles University, 12108 Prague, Czech Republic; david.krepelka@lf1.cuni.cz (D.K.); ales.dvorak@lf1.cuni.cz (A.D.); vitek@cesnet.cz (L.V.); 5 Department of Medical, Surgical, and Health Sciences, University of Trieste, 34127 Trieste, Italy; cbottin@units.it (C.B.); f.zanconati@fmc.units.it (F.Z.); 6 Cognitive Neuroscience Department, International School for Advanced Studies (SISSA), Via Bonomea, 265, 34136 Trieste, Italy; aufrance@sissa.it; 7 Department of Medical Laboratory Science, Hebron University, Hebron P785, Palestine; qaisiyam@hebron.edu; 8 4th Department of Internal Medicine, General University Hospital in Prague, 1st Faculty of Medicine, Charles University, 12108 Prague, Czech Republic

**Keywords:** Grm1, PKC3, calcium, kernicterus spectrum disorder, gene clustering, Gunn rats, neurotoxicity, glutamate receptors, dystonia, neonatal jaundice

## Abstract

Kernicterus spectrum disorder is the permanent and highly disabling neurologic sequel of neonatal exposure to hyperbilirubinemia, presenting, among other symptoms, variable and untreatable motor disabilities. To search for potential biomolecular explanations, we used a Gunn rat colony exhibiting spontaneous hyperbilirubinemia and a large variability of motor deficits on a beam-walking test. Histological and microscopic analyses confirmed worsening damage in the cerebellum (Cll; hypoplasia, increased death of neurons, and disrupted astroglial structures) and parietal motor cortex (hCtx; increased cell sufferance and astrogliosis). Clustering and network analyses of transcriptomic data reveal rearrangement of the physiological expression patterns and signaling pathways associated with bilirubin neurotoxicity. Bilirubin content among hyperbilirubinemic (jj) animals is overlapped, which suggests that the amount of bilirubin challenge does not fully explain the tissue, transcriptomic, proteomic, and neurobehavioral alterations. The expression of nine genes involved in key postnatal brain development processes is permanently altered in a phenotype-dependent manner. Among them, Grm1, a metabotropic glutamatergic receptor involved in glutamate neurotoxicity, is consistently downregulated in both brain regions both at the transcriptomic and proteomic levels. Our results support the role of Grm1 and glutamate as biomolecular markers of ongoing bilirubin neurotoxicity, suggesting the possibility to improve diagnosis by ^1^H-MR spectroscopy.

## 1. Introduction

Severe neonatal hyperbilirubinemia, if not promptly diagnosed and managed, may lead to neurological deficits with large variability and severity in both its signs and symptoms [1,2,3,4,5,6,7].

In the first three to five days (early phase), severely hyperbilirubinemic neonates exhibit mild lethargy, poor feeding and sucking, slight hypotonia and hyperreflexia, and a slightly high-pitched cry, which are generally reversible. At the end of the first week, the infants’ conditions can progress into two phases: intermediate and late/advanced phases. Symptoms would include moderate to deep stupor, irritability, fever, alternating hypotonia and hypertonia, back arching, pronounced-to-severe muscle hyperextension (retrocollis–opisthotonos), inability to feed, and high-pitched crying, as well as hearing and visual abnormalities, athetosis, seizures, and pronounced hypotonia. These two phases may either be reversible or lead to kernicterus spectrum disorder (KSD) or death [1,8,9,10,11,12,13,14,15,16].

The KSD classification is primarily based on the severity of motor and auditory impairments, supplemented with hearing aids. Motor signs and symptoms include mild gross motor delays and mild dystonia with or without athetosis, moderate hyperkinetic dystonia or ‘athetoid cerebral palsy (CP)’ (ambulates with or without assistance), and severe dystonia/hyperkinetic CP (patients unable to ambulate, feed self, sign or speak; often with muscle cramps and episodic severe hypertonia throughout childhood and adulthood) [2,17,18]. In 2010, Shapiro et al. captured the picture of this condition’s variability with the term kernicterus spectrum disorder (KSD) [17]. Unfortunately, the biomolecular reasons for this variability are still largely unexplained.

In our previous work, we investigated the possible mechanism that underlies bilirubin neurotoxicity, reporting a bilirubin-induced alteration of genes involved in postnatal brain development and a transcriptomic imprinting of a panel of genes involved mainly in synaptogenesis and glutamatergic neurotransmission in the cerebral parietal cortex (designated as hCtx, a sensory–motor region) of hyperbilirubinemic (jj) Gunn rats. These mechanisms agree with the available clinical information (discussed in [19]). Together with the hCtx, the cerebellum (Cll), another region of the brain involved in motor control, is well known to be permanently and macroscopically damaged in a rodent model of neonatal hyperbilirubinemia [20,21,22,23,24].

In this study, we used our Gunn rat colony exhibiting large variability in motor behavior as hyperbilirubinemic (jj) animals compared to their normobilirubinemic (NJ) littermates. Hyperbilirubinemia (jj) presents either a severe phenotype (SP) with frank behavioral abnormalities (tremors, balance deficits, dystonia, wobbly gait; lately SP: severe phenotype) or low-phenotype (LP) jj rats with deficits detectable only by tests—to depict the possible biomolecular alterations causing the worsening motor functions. Following the worsening of the phenotype, we quantified the motor performances, the bilirubin concentrations in the blood and brain, the progression of histological damage, and the transcriptomic and proteomic changes of selected targets [19,20,24]. Finally, we identified the most notable targets to pursue at the proteomic level, assessed alterations in the cluster of expressions, and performed correlational analysis between gene expression and the phenotype.

## 2. Results

### 2.1. Evident Severe Motor Deficits in Severe-Phenotype (SP) Gunn Rats

#### 2.1.1. Beam-Walking Test

**Figure 1:**
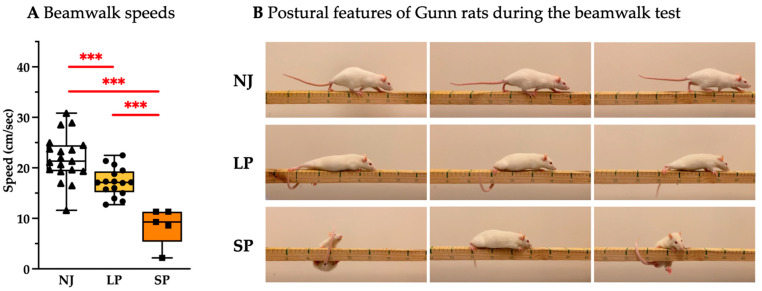
Behavioral assessment of coordination, balance, and speed of adult NJ, LP, and SP Gunn rats. (**A**) Quantification of beam-walking performance between NJ, LP, and SP rats. Results are presented as median and min/max of 5–19 animals per group. Each dot in the plots = 1 animal. Statistical significance: *** *p* < 0.001. (**B**) Representative photos showing the postural features of NJ, LP, and SP rats. NJ—normobilirubinemic Gunn rats (triangle symbols, white box); LP—low-phenotype hyperbilirubinemic Gunn rats (circle symbols, yellow box); SP—severe- phenotype hyperbilirubinemic Gunn rats (square symbols, orange box).

To assess rodents’ fine motor coordination and balance, a beam-walking test was performed. Figure 1A depicts the significantly worse performance of severe-phenotype (SP) animals versus the low phenotype (LP) (*p* < 0.001) and the normobilirubinemic (NJ) control group (*p* < 0.001, with LP *p* < 0.001 vs. NJ). Figure 1B highlights the declining motor abilities such as difficulty in walking with four limbs and lifting their bodies (LP and SP). Phenotypically, SP rats showed abnormal postures on the beam and balance problems, which were represented by falling off the beam while trying to traverse, and some were unable to start and complete the test. Although LP rats can traverse the full length of the beam, they show abnormal gaits where they appear to drag their hind limbs through the beam with the abdomen closer to the axis. The NJ animals exhibited good balance and locomotion, fully lifting themselves using all their limbs as they fully traversed the wooden beam.

#### 2.1.2. Cerebellar Hypoplasia

**Figure 2:**
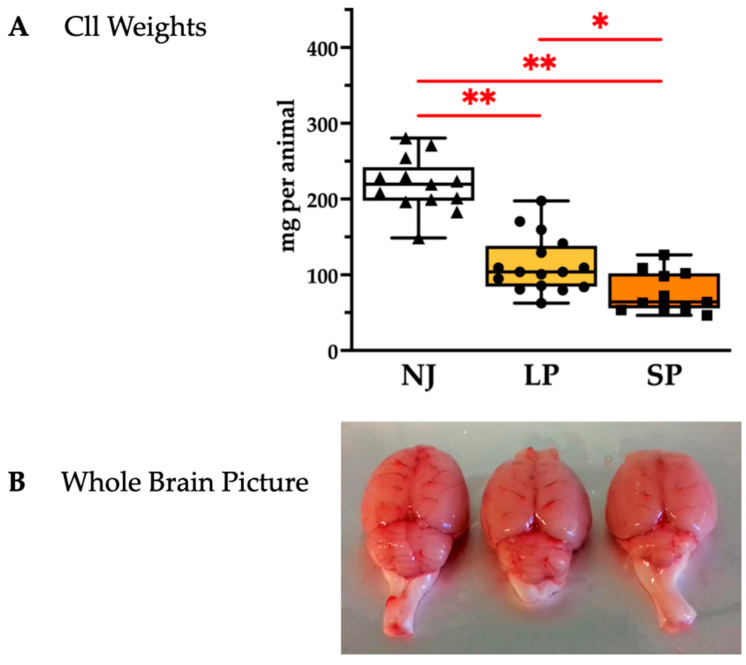
Cerebellar characterization of NJ, LP, and SP Gunn rats. (**A**) Cerebellum weights presented as median and min/max of (11–16) animals per group; each dot in the plots = 1 animal. Statistical significance: * *p* < 0.05; ** *p* < 0.01. (**B**) Representative photos showing an evident worsening of cerebellar hypoplasia in SP. NJ—normobilirubinemic Gunn rats (triangle symbols, white box); LP—low-phenotype hyperbilirubinemic Gunn rats (circle symbols, yellow box); SP—severe-phenotype hyperbilirubinemic Gunn rats (square symbols, orange box); Cll—cerebellum.

To assess cerebellar hypoplasia, a hallmark feature of jj Gunn rats [20,21,23,24], we quantified the weight of the Cll (sizes of Cll depicted in Figure 2A and quantified in Figure 2B). SP rats exhibited the worst Cll hypoplasia with a further 30% decrease in Cll vs. LP (*p* < 0.05; SP vs. NJ: *p* < 0.01), while LP rats had a 50% decrease in Cll weight vs. NJ (*p* < 0.01).

**Figure 3:**
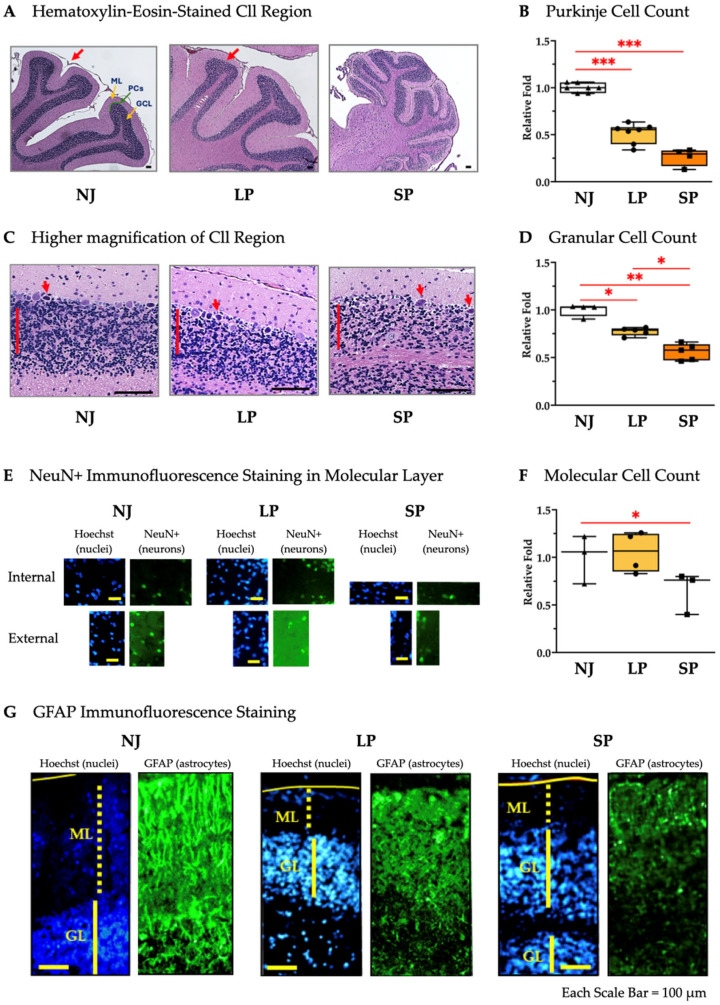
Histological and cellular characterization of Cll in NJ, LP, and SP Gunn rats. (**A**) Hematoxylin–eosin-stained cerebellar regions of NJ, LP, and SP showing the folia and extensions (red arrows). Scale bar = 100 µm. (**B**) Purkinje cell counts in NJ, LP, and SP. Data are median and min/max of (4–7) animals per phenotype. Statistical significance: *** *p* < 0.001. (**C**) Higher magnification of the cerebella of NJ, LP and SP showing the density of the granular cell layer (red lines) and suffering PCs (red arrows). Scale bar = 100 µm. (**D**) Granular cell counts in the cerebella of NJ, LP, and SP. Data are median and min/max of (4–5) animals per phenotype. Statistical significance: * *p* < 0.05; ** *p* < 0.01. (**E**) Immunofluorescence staining of non-granular neurons in the molecular layer of the Cll with NeuN in NJ, LP, and SP. Scale bar = 100 µm. (**F**) NeuN^+^ cell counts in the molecular layers of the Cll of NJ, LP, and SP. Data are median and min/max of (3–4) animals for each phenotype. Statistical significance: * *p* < 0.05. (**G**) Immunofluorescence staining of astrocytes with GFAP in NJ, LP, and SP. Scale bar = 100 µm. NJ—normobilirubinemic Gunn rats (triangle symbols, white box); LP—low-phenotype hyperbilirubinemic Gunn rats (circle symbols, yellow box); SP—severe-phenotype hyperbilirubinemic Gunn rats (square symbols, orange box); Cll—cerebellum; NeuN: Neuronal Nuclear Antigen and Neuron Differentiation Marker; GFAP—glial fibrillary acidic protein; ML: molecular layer; GL: granular cell layer; PCs: Purkinje cells. (**B**,**D**,**F**) Each dot in the plots = 1 animal.

Cll hypoplasia was further characterized through histological analysis of the sagittal brain sections. Figure 3 shows the hematoxylin–eosin-stained Cll regions of NJ, LP, and SP. The number of the folia and their extensions observable in Cll of NJ at this magnification is few, as it has the largest area, suggesting well-developed Cll features, while LP exhibits an under-developed architecture that is further aggravated in SP (Figure 3A, red arrows). Under the same magnification, the whole Cll of SP is fully captured compared to the representative Cll regions of NJ and LP (Figure 3A). Furthermore, Purkinje cell (PC) counts revealed that LP and SP have a 1.94-fold (*p* < 0.001) and a 3.79-fold reduction (*p* < 0.001) versus NJ, respectively (Figure 3B). Higher magnification (Figure 3C) allowed better visualization of the ongoing death of PCs (red arrows) as well as the visible reduction in density of the granular layers (indicated by red lines in Figure 3C) in LP and SP. The number of neurons in the granular layer were significantly reduced in SP rats (1.79-fold reduction), followed by LP (1.29-fold reduction) compared to NJ rats (Figure 3D). The number of neurons in the molecular layer (Figure 3E) was found to be decreased in SP (*p* < 0.05) while the number of neurons is comparable between NJ and LP (Figure 3F).

GFAP staining revealed the partial disruption in LP and the complete disruption of astroglial structures in SP (Figure 3F).

In general, these histological results including the counts of neurons (PCs, granular and NeuN^+^ cells), as well as GFAP signal alterations, confirm that SP rats have more severe Cll damage than their LP counterparts.

#### 2.1.3. Parietal Motor Cortex Damage at the Microscopic Level

**Figure 4:**
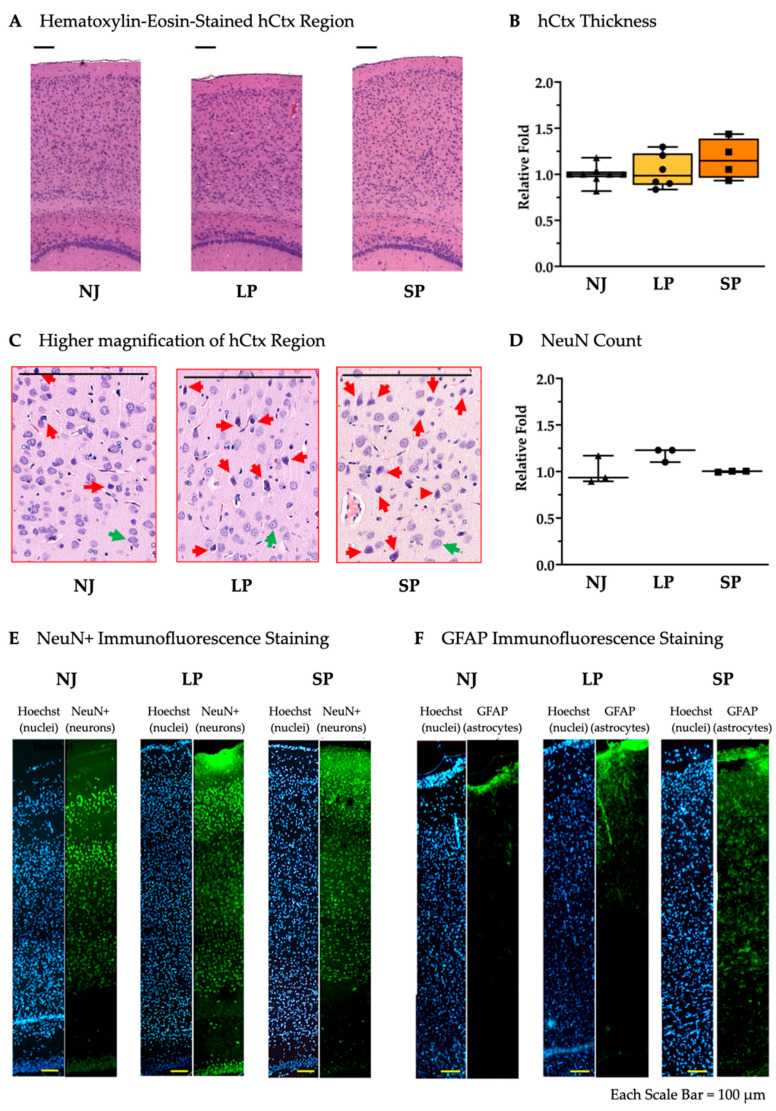
Histological and cellular characterization of hCtx in NJ, LP, and SP Gunn rats. (**A**) Hematoxylin–eosin staining of the hCtx regions in NJ, LP and SP. Scale bar = 100 µm. (**B**) Relative hCtx thickness not significantly different in NJ, LP, and SP. Data are median and min/max of (4–8) animals per phenotype. (**C**) Higher magnification of hematoxylin–eosin-stained hCtx in NJ, LP, and SP. (red arrows = suffering cells; green arrows healthy cells). Scale bar = 100 µm. (**D**) NeuN^+^ cell count in the hCtx of NJ, LP, and SP. Data are median and min/max of 3 animals per phenotype. (**E**) Immunofluorescent staining of neurons with NeuN in hCtx from NJ, LP, and SP. (**F**) Immunofluorescent staining of astrocytes with GFAP in the hCtx of NJ, LP, and SP. NJ (white box; triangle symbols)—normobilirubinemic Gunn rats; LP (yellow box; circle symbols)—low-phenotype hyperbilirubinemic Gunn rats; SP (orange box; square symbols)—severe-phenotype hyperbilirubinemic Gunn rats; hCtx—parietal motor cortex; GFAP—glial fibrillary acidic protein. (**B**,**D**) Each dot in the plots = 1 animal.

To assess the presence of any bilirubin-induced damage in the hCtx, we measured its thickness among NJ, LP, and SP and found them comparable with no significant differences (Figure 4A,B). Despite the fact that hyperbilirubinemic rats exhibited suffering cells—large, deeply colored cells without defined nuclei and cytoplasmatic regions (Figure 4C, red arrows) compared to the healthy cells (Figure 4C, green arrows)—the quantification of NeuN^+^ cells revealed no neuronal loss (Figure 4D,E), while a frank astrogliosis was present in SP compared to LP and NJ (Figure 4F).

These results reveal the ongoing bilirubin toxicity due to the presence of astrogliosis even in the absence of macroscopic damage in hCtx.

### 2.2. Genes Associated with Synaptic Plasticity, Cell Proliferation and Differentiation, and Neuronal Development Are Significantly Modulated in SP Animals and Are Correlated with Motor Performance

**Figure 5:**
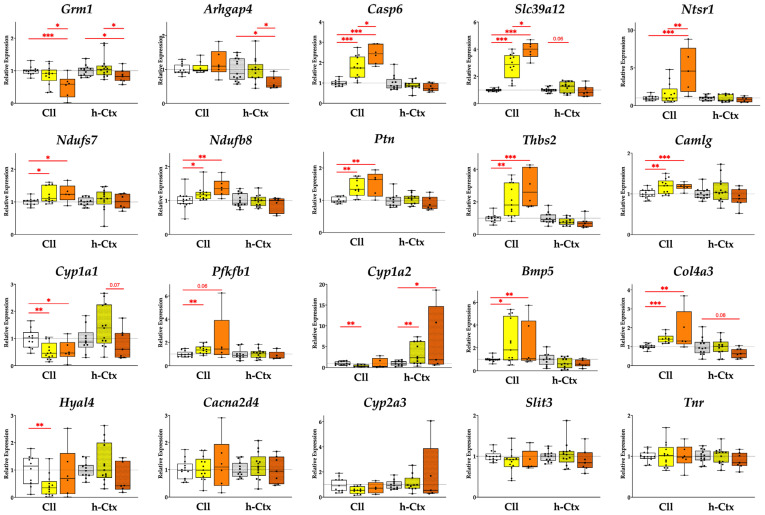
Expression of relevant genes in the Cll and hCtx. Five genes are significantly modulated in SP vs. LP while some genes exhibit a phenotype-dependent pattern of expression across the different groups. Results presented as median and min–max of (4–13) animals per group. * *p* < 0.05; ** *p* < 0.01; *** *p* < 0.001. Each dot in the plots = 1 animal. NJ—normobilirubinemic Gunn rats (white boxes); LP—low-phenotype hyperbilirubinemic Gunn rats (yellow boxes); SP—severe-phenotype hyperbilirubinemic Gunn rats (orange boxes); Cll—cerebellum; hCtx—parietal motor cortex; *Arhgap4*—Rho-GTPase-activating protein 4; *Bmp5*—bone morphogenetic protein 5; *Cacna2d4*—calcium voltage-dependent calcium channel complex alpha-2/delta subunit family; *Camlg*—calcium modulating ligand; *Casp6*—caspase 6; *Col4a3*—collagenase 4a3; *Cyp1a1/1a2/2a3*—cytochrome P450 1a1/1a2/2a3; *Grm1*—glutamate metabotropic receptor 1; *Hyal4*—hyaluronic acid 4; *Ndufs7/b8*: NADH—ubiquinone oxidoreductase (complex I) subunit 7/8; *Ntsr1*—neurotensin receptor 1; *Pfkfb1*—6-phosphofructo-2-kinase/fructose-2,6-biphosphatase 1; *Ptn*—pleiotrophin; *Slc39a12*—solute carrier family 39 member 12; *Slit3*—slit guidance ligand 3; *Thbs2*—thrombospondin 2; *Tnr*—tenascin R.

Twenty (20) genes that are known to be permanently altered in adult jj vs. NJ rats [19,20,24] were also evaluated in our study (Table 1 and Figure 5).

As a priority, we highlight genes that are significantly modulated in SP vs. LP, followed by genes that are significantly modulated in SP vs. NJ, especially those that show a phenotype-dependent trend of gene expressions from NJ through LP to SP.

#### 2.2.1. Genes Significantly Modulated in SP vs. LP

SP vs. LP in either the Cll or hCtx region showed significant modulation in five genes examined (Figure 5, first row).

*Grm1* (glutamatergic metabotropic receptor 1) was significantly downregulated in SP vs. LP (1.65-fold, *p* ≤ 0.05 in Cll and 1.31-fold *p* ≤ 0.05 in hCtx) and SP vs. NJ (1.92-fold, *p* ≤ 0.001 in Cll and 1.19-fold, *p* ≤ 0.05 in hCtx).

*Arhgap4* (Rho GTPase-activating protein 4) expression was significantly reduced in the hCtx of SP vs. LP (1.6-fold *p* ≤ 0.05) and vs. NJ (1.64-fold *p* ≤ 0.05). In the Cll, Arhgap4 expression was generally increased in SP (SP vs. LP = 1.14-fold increase; SP vs. NJ = 1.19-fold increase; LP vs. NJ = 1.05-fold increase) but the difference was not statistically significant.

*Casp6* (caspase 6) expression had a significant upregulation in the Cll region of SP (vs. LP = 1.37-fold increase, *p* ≤ 0.05; SP vs. NJ = 2.42-fold increase, *p* ≤ 0.001) and LP (vs. NJ = 1.77-fold increase, *p* ≤ 0.001) rats. In hCtx, Casp6 expression was generally decreased in hyperbilirubinemic animals (SP vs. LP = 1.14-fold decrease; SP vs. NJ = 1.29-fold decrease; LP vs. NJ = 1.13-fold decrease), although the differences were not statistically significant.

*Slc39a12* (solute carrier family 39 member 12, or Zip12) had a significant upregulation in the Cll region of SP (vs. LP = 1.42-fold increase, *p* ≤ 0.01; SP vs. NJ = 3.94-fold increase, *p* ≤ 0.001) and LP (vs. NJ = 2.79-fold increase, *p* ≤ 0.01). In hCtx, *Slc39a12* expression was generally decreased in SP (vs. LP = 1.14-fold decrease; SP vs. NJ = 1.29-fold decrease), but increased in LP (vs. NJ = 1.23-fold increase). However, these differences were not statistically significant in the hCtx.

*Ntsr1* (neurotensin receptor 1) was significantly upregulated in the Cll region of SP (vs. LP = 2.94-fold increase, *p* ≤ 0.01; SP vs. NJ = 4.7-fold increase, *p* ≤ 0.001) and LP (vs. NJ = 1.6-fold increase, *p* ≤ 0.01). In the hCtx, *Ntsr1* expression was generally decreased in SP (vs. LP = 1.23-fold decrease; SP vs. NJ = 1.26-fold decrease; LP vs. NJ = 1.03-fold increase). However, these differences were not statistically significant in the hCtx.

#### 2.2.2. Genes Expressed in SP with Statistical Difference vs. NJ Only and Genes Expressed with Consistent Trends Along the Worsening Phenotypes

Moving to the second priority, expressions of *Ndufs7* and *Ndufb8* (Cll, *p* < 0.05 and *p* < 0.01; NADH—ubiquinone oxidoreductase (complex I) subunit 7/8), *Ptn* (Cll, *p* < 0.01; pleiotrophin), and *Thbs2* (Cll, *p* < 0.001; thrombospondin 2) showed an increasing trend reaching statistical significance in SP vs. NJ in Cll.

#### 2.2.3. Other Genes

The last group of genes was either not altered among the different groups or similarly modulated between LP and SP, or SP reverted to the NJ level, suggesting that they may not be relevant in the worsening of the phenotype.

The expressions of *Camlg* (Cll, *p* < 0.001; calcium modulating ligand), *Cyp1a1* (Cll, *p* < 0.05; cytochrome P450), *Cyp1a2* (hCtx, *p* < 0.05), *Bmp5* (Cll, *p* < 0.05; bone morphogenetic protein 5), and *Col4a3* (Cll, *p* < 0.01; collagenase 4a3) in SP are statistically different vs. NJ. Meanwhile, *Hyal4* (hyaluronic acid 4), *Cacna2d4* (calcium voltage-dependent calcium channel complex alpha-2/delta subunit family), *Cyp2a3*, *Slit3* (slit guidance ligand 3), and *Tnr* (tenascin R) showed no modulation.

#### 2.2.4. Gene Correlations with Motor Performance

**Figure 6:**
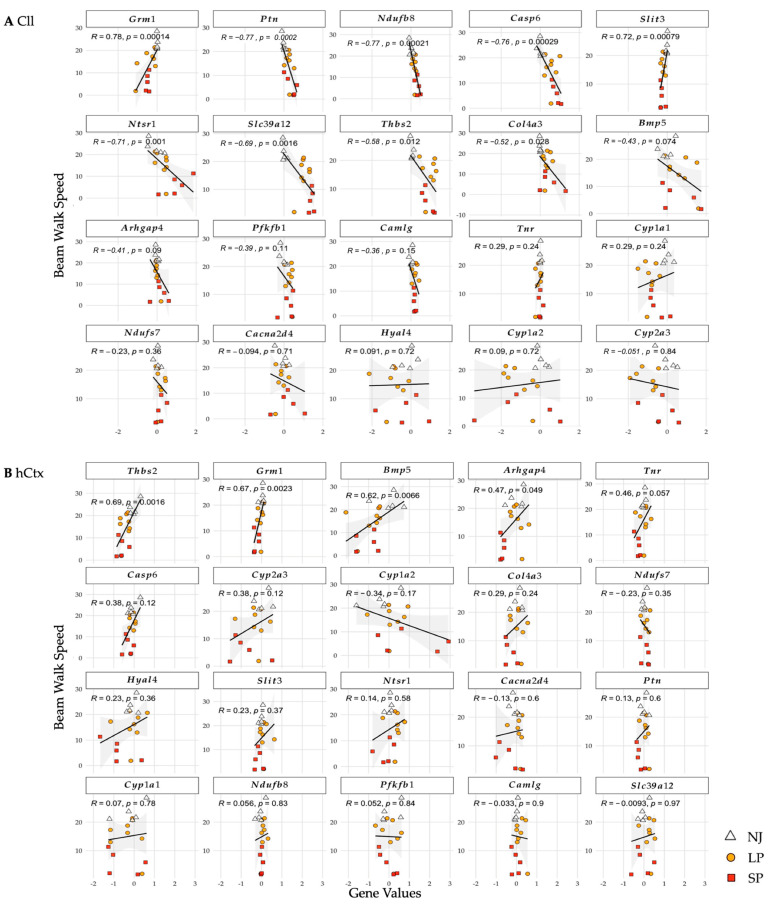
Correlation of genes and beam-walking performance in Cll (A) and hCtx (B). R values (correlation coefficients; value closer to 1 means stronger correlation) and *p*-values (statistical significance of the correlation). Each dot = 1 animal. NJ—normobilirubinemic (white triangles); LP—low-phenotype hyperbilirubinemic (yellow circles); SP—severe-phenotype hyperbilirubinemic (orange squares); Cll—cerebellum; hCtx—parietal motor cortex; *Arhgap4*—Rho-GTPase-activating protein 4; *Bmp5*—bone morphogenetic protein 5; *Cacna2d4*—calcium voltage-dependent calcium channel complex alpha-2/delta subunit family; *Camlg*—calcium modulating ligand; *Casp6*—caspase 6; *Col4a3*—collagenase 4a3; *Cyp1a1/1a2/2a3*—cytochrome P450 1a1/1a2/2a3; *Grm1*—glutamate metabotropic receptor 1; *Hyal4*—hyaluronic acid 4; *Ndufs7/b8*: NADH—ubiquinone oxidoreductase (complex I) subunit 7/8; *Ntsr1*—neurotensin receptor 1; *Pfkfb1*—6-phosphofructo-2-kinase/fructose-2,6-biphosphatase 1; *Ptn*—pleiotrophin; *Slc39a12*—solute carrier family 39 member 12; *Slit3*—slit guidance ligand 3; *Thbs2*—thrombospondin 2; *Tnr*—tenascin R.

To further link gene modulation with behavior, the correlation between gene expressions and motor performance was also analyzed (Figure 6).

In the Cll (Figure 6A), statistically significant negative correlations (all *p* ≤ 0.05, decreased speed = increased mRNA level) were shown for *Casp6*, *Slc39a12*, *Ntsr1*, *Ndufb8*, *Ptn*, *Thbs2*, and *Col4a3*. On the contrary, *Grm1* and *Slit3* showed strong positive correlations (decreased speed = decreased mRNA expression). In the hCtx (Figure 6B), statistically significant positive correlations were shown in *Grm1*, *Arhgap4*, *Thbs2*, and *Bmp5*.

These results suggest that upregulation of *Casp6*, *Slc39a12*, *Ntsr1*, *Ndufb8*, *Ptn*, *Thbs2*, and *Col4a3* and downregulation of *Grm1*, *Arhgap4*, *Thbs2*, *Bmp5*, and *Slit3* in the two brain motor regions of Gunn rats are correlated with motor dysfunctions.

Among all these genes, only *Grm1* was consistently downregulated in SP compared to LP and NJ in both the Cll and hCtx regions and was strongly correlated with worsening of the motor phenotype.

#### 2.2.5. Gene Clustering Analysis

To identify similar patterns of mRNA expression and their changes in LP and SP, hierarchical clustering analysis was performed. Similar expression patterns between genes are shown by clustering via branches. The lower the distance between branches, the higher the similarity.

**Figure 7:**
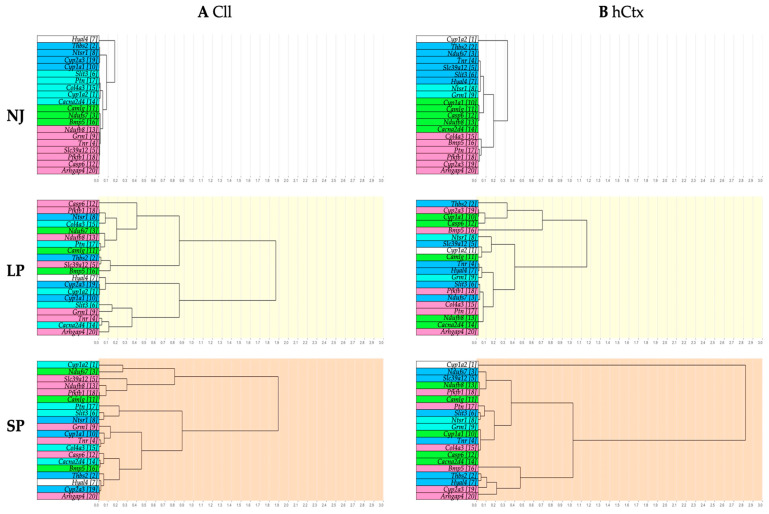
Dendrograms showing hierarchical gene clustering in Cll (**A**) and hCtx (**B**). NJ (white background)—normobilirubinemic Gunn rats; LP (yellow background)—low-phenotype hyperbilirubinemic Gunn rats; SP (orange background)—severe-phenotype hyperbilirubinemic Gunn rats; Cll—cerebellum; hCtx—parietal motor cortex; blue, cyan, green and pink: colors identifying the original clusters in NJ animals. Each gene in NJ rats is additionally identified by a sequential number to help in following the rearrangement of gene clustering in LP and SP animals. *Arhgap4*—Rho-GTPase-activating protein 4; *Bmp5*—bone morphogenetic protein 5; *Cacna2d4*—calcium voltage-dependent calcium channel complex alpha-2/delta subunit family; *Camlg*—calcium modulating ligand; *Casp6*—caspase 6; *Col4a3*—collagenase 4a3; *Cyp1a1/1a2/2a3*—cytochrome P450 1a1/1a2/2a3; *Grm1*—glutamate metabotropic receptor 1; *Hyal4*—hyaluronic acid 4; *Ndufs7/b8*: NADH—ubiquinone oxidoreductase (complex I) subunit 7/8; *Ntsr1*—neurotensin receptor 1; *Pfkfb1*—6-phosphofructo-2-kinase/fructose-2,6-biphosphatase 1; *Ptn*—pleiotrophin; *Slc39a12*—solute carrier family 39 member 12; *Slit3*—slit guidance ligand 3; *Thbs2*—thrombospondin 2; *Tnr*—tenascin R.

The NJ group (Figure 7, dendrogram with a white background as physiological control) shows the four main gene clusters in the Cll (blue: *Thbs2*, *Ntsr1*, *Cyp2a3*, *Cyp1a1*; cyan: *Slit3*, *Ptn*, *Col4a3*, *Cyp1a2*, *Cacna2d4*; green: *Camlg*, *Ndufs7*, *Bmp5*; pink: *Ndufb8*, *Grm1*, *Tnr*, *Slc39a12*, *Pfkfb1*, *Casp6*, *Arhgap4*) (Figure 7A) and four main gene clusters in the hCtx (blue: *Thbs2*, *Ndufs7*, *Tnr*, *Slc39a12*, *Slit3*, *Hyal4*; cyan: *Ntsr1*, *Grm1*; green: *Cyp1a1*, *Camlg*, *Casp6*, *Ndufb8*, *Cacna2d4*; pink: *Col4a3*, *Bmp5*, *Ptn*, *Pfkfb1*, *Cyp2a3*, *Arhgap4*) (Figure 7B). It should be noted that the gene hierarchy (assigned numbering of genes in hCtx assigned as reference, Figure 7B—NJ) is different between Cll and hCtx.

When observing SP and LP gene clusters, there is an obvious rearrangement (follow the background colors). This indicates a disruption between physiologically co-expressed genes. Moreover, the rearrangement of the gene among LP and SP is minimal in the Cll and much higher in hCtx. This might suggest (1) a different response to bilirubin among different brain regions [19,20,89,90,91,92,93,94,95]; (2) that the profile of gene expressions in Cll is mostly driven by bilirubin and does not further identify the LP or SP phenotype; and (3) that in the hCtx region, the extent of changes between SP and LP better parallels changes in motor functions, despite the absence of macroscopic histologic damage. The third point may suggest that classic MRI approaches may have enough sensitivity and/or definition to identify the effects of bilirubin toxicity.

#### 2.2.6. Gene Network Analysis

**Figure 8:**
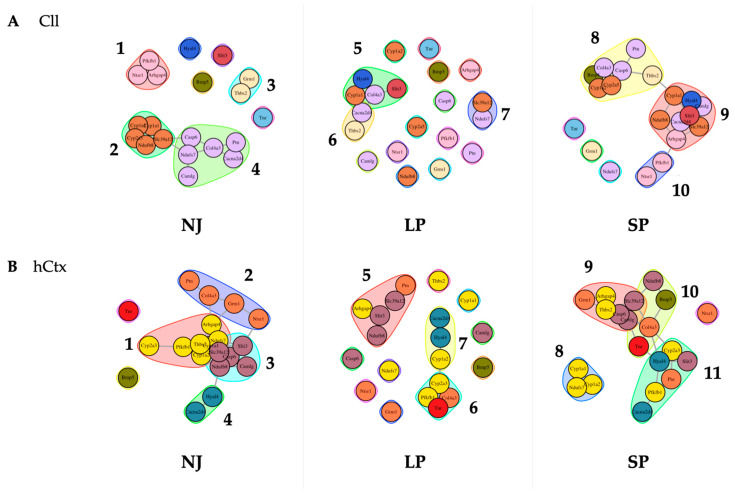
Predicted gene functional networks between phenotypes NJ, LP, and SP. Perturbation of physiological clusters (NJ) highlights the significant effect on the signaling pathways and biomolecular processes associated with the expression of genes in the Cll (**A**) and the hCtx (**B**) of hyperbilirubinemic animals. NJ—normobilirubinemic Gunn rats; LP—low-phenotype hyperbilirubinemic Gunn rats; SP—severe-phenotype hyperbilirubinemic Gunn rats; Cll—cerebellum; hCtx—parietal motor cortex; *Arhgap4*—Rho-GTPase-activating protein 4; *Bmp5*—bone morphogenetic protein 5; *Cacna2d4*—calcium voltage-dependent calcium channel complex alpha-2/delta subunit family; *Camlg*—calcium modulating ligand; *Casp6*—caspase 6; *Col4a3*—collagenase 4a3; *Cyp1a1/1a2/2a3*—cytochrome P450 1a1/1a2/2a3; *Grm1*—glutamate metabotropic receptor 1; *Hyal4*—hyaluronic acid 4; *Ndufs7/b8*: NADH—ubiquinone oxidoreductase (complex I) subunit 7/8; *Ntsr1*—neurotensin receptor 1; *Pfkfb1*—6-phosphofructo-2-kinase/fructose-2,6-biphosphatase 1; *Ptn*—pleiotrophin; *Slc39a12*—solute carrier family 39 member 12; *Slit3*—slit guidance ligand 3; *Thbs2*—thrombospondin 2; *Tnr*—tenascin R.

To further assess how changes in gene expression contribute to the severity of motor phenotype, gene network analysis was performed. This bioinformatic approach generated a new panel of clusters by correlating the level of mRNA expression to a list of potential biological functions, signaling pathways, and diseases. Functional clusters are depicted in Figure 8, with all genes belonging to a physiological cluster (NJ) identified by a color, again to help visualize the rearrangements along LP and SP. The full list is of potential biological functions, signaling pathways, and diseases identified for each cluster is presented in Tables S3 and S4; those of which have some background in the bilirubin literature are in red text.

In Cll (Figure 8A), there are four clusters in NJ (physiological control group):

Cluster 1 (*Arhgap4*, *Ntsr1*, *Pfkfb1*) and Cluster 3 (*Grm1*, *Thbs2*) show no association with the other genes or clusters, while Cluster 2 (*Cyp1a1*, *Cyp1a2*, *Cyp2a3* [in rats, *Cyp2a5* in humans], *Ndufb8*, *Slc39a12*) is linked to Cluster 4 (*Casp6*, *Ndufs7*, *Camlg*, *Col4a3*, *Cacna2d4*, *Ptn*) via *Slc39a12*, *Casp6* and *Ndufs7.* Only *Slit3* and *Tnr*, *Hyal4*, and *Bmp5* are the non-clustered genes.

These physiological associations are disrupted in LP, with the generation of Cluster 5 (*Slit3*, *Hyal4*, *Cyp1a1*, *Col4a3*) interconnected with Cluster 6 (*Cacna2d4*, *Thbs2*), and an independent Cluster 7 (*Slc39a12*, *Ndufs7*). Most evidently, there are 12 non-associated genes: *Arhgap4*, *Bmp5*, *Camlg*, *Casp6*, *Cyp1a2*, *Cyp2a3*, *Grm1*, *Ndufb8*, *Ntsr1*, *Pfkfb1*, *Ptn* and *Tnr*. Notably, based on the results of the gene network analysis, the rearrangement introduced new potential biological activities, including some already known in bilirubin neurotoxicity, e.g., apoptosis, long-term depression and potentiation [96,97,98], glutamatergic synapse [63], and calcium signaling pathway activity [99,100,101], as well as association with multiple diseases (Table S3), whose biological importance and real relevance to the disease have yet to be further understood/evaluated.

Most of these non-associated genes in the LP group have re-associated in the SP into three different interconnected clusters: Cluster 8 (*Bmp5*, *Cyp1a2*, *Cyp2a5*, *Col4a3*, *Casp6*, *Ptn*, *Thbs2*) interconnected via *Thbs2* with *Cyp1a1* of Cluster 9 (*Cyp1a1*, *Ndufb8*, *Slc39a12*, *Hyal4*, *Slit3*, *Camlg*, *Cacna2d4*, *Arhgap4*), which is interconnected via *Arhgap4* with *Pfkfb1* of Cluster 10 (*Pfkfb1*, *Ntsr1*). Only three genes are non-associated, namely *Grm1*, *Ndufs7*, and *Tnr*.

All these clusters in Cll in SP are highly different compared to physiological clusters (NJ) and clusters in LP rats. Nevertheless, the most known mechanisms of bilirubin-induced toxicity (e.g., long-term depression and potentiation [96,97,98], glutamatergic synapse [63]) are confirmed in SP rats (see red text in Table S3).

In the hCtx of the physiologic controls, there are four interconnected clusters (Figure 8B):

Cluster 1 (*Cyp2a3*, *Pfkfb1*, *Thbs2*, *Cypa1a2*, *Ndufs7*, *Arhgap4*—ROS, metabolism of xenobiotics by CYP, DNA adducts, receptor activation [102,103]) is interconnected via *Arhgap4* with *Col4a3* and *Grm1* of Cluster 2 (*Ptn*, *Col4a3*, *Grm1*, *Ntsr1*—calcium signaling pathway [99,100,101]) which is interconnected via *Ntsr1* with *Slit3* of Cluster 3 (*Slit3*, *Slc39a12*, *Casp6*, *Camlg*, *Ndufb8*); *Ndufb8* interconnects Cluster 3 with *Hyal4* of Cluster 4 (*Hyal4*, *Cacna2d4*). Notably, Cluster 1 and Cluster 3 are intricately interconnected among *Thbs2*, *Ndufs7*, *Cyp1a2*, and *Cyp1a1* and *Slc39a12*, *Ndufb8*, and *Casp6*. There are only two non-associated genes, *Bmp5* and *Tnr*.

Most of these physiological associations are also disrupted in LP: an independent Cluster 5 (*Arhgap4*, *Slit3*, *Ndufb8*, *Slc39a12*, *Ptn*), and interconnected Cluster 6 (*Tnr*, *Pfkfb1*, *Col4a3*, *Cyp2a3*—ECM–receptor interaction, PI3K-Akt signaling pathway [104]) and Cluster 7 (*Cyp1a2*, *Hyal4*, *Cacna2d4*—drug and xenobiotic metabolism via CYP [103], DNA adducts [102]) via *Cyp1a2* and *Cyp2a3*. There are eight non-associated genes: *Casp6*, *Ntsr1*, *Ndufs7*, *Grm1*, *Thbs2*, *Cyp1a1*, *Camlg*, and *Bmp5*.

In the SP group, there are four clusters: Cluster 9 (*Grm1*, *Arhgap4*, *Thbs2*, *Casp6*, *Slc39a12*, *Camlg*) via *Camlg* and *Slc39a12* interconnected with the genes *Tnr*, *Col4a3* and *Ndufb8* of Cluster 10 (*Ndufb8*, *Bmp5*, *Col4a3*, *Tnr*—ECM–receptor interaction, PI3K-Akt signaling pathway [104]); Cluster 10 interconnected via *Tnr* and *Col4a3* with *Cyp2a3* and *Hyal4* of Cluster 11 (*Cyp2a3*, *Hyal4*, *Slit3*, *Ptn*, *Pfkfb1*, *Cacna2d4*); and an independent Cluster 8 (*Cyp1a1*, *Cyp1a2*, *Ndufs7*—ROS, metabolism by xenobiotics by CYP [103], DNA adducts [102]). There is only one non-associated gene, *Ntsr1* (calcium signaling pathway [99,100,101]).

Overall, the results of the gene network analysis (1) confirm the differences between Cll and hCtx already present in NJ (physiological differences), (2) highlight a differential response to bilirubin challenge in LP and SP rats, and (3) point to the alteration of synapse-related functions (e.g., apoptosis, calcium signaling pathway, cytoskeleton in muscle cells, oxidative phosphorylation), particularly of glutamatergic type (e.g., glutamate synapses, long-term potentiation and depression) (see Tables S3 and S4).

### 2.3. Proteomic Analysis Confirms Significant Modulations in the Synaptic Plasticity, Cell Proliferation and Differentiation, and Neuronal Development of SP Animals

Four targets (Grm1, Arhgap4, Ntsr1, Slc39a12) were further investigated at the proteomic level based on the transcriptomic data showing a significant modulation in SP vs. LP.

**Figure 9:**
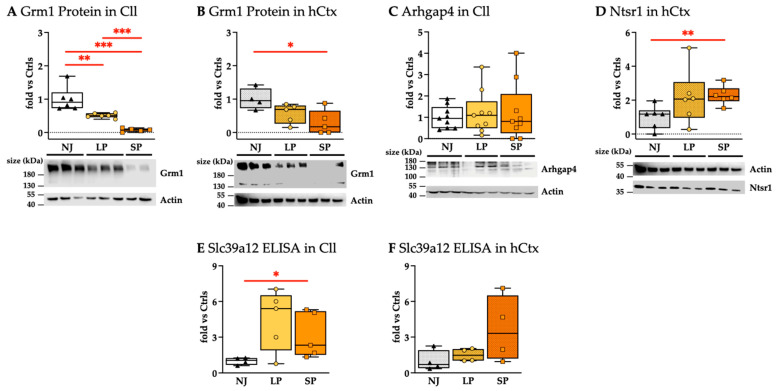
Protein analysis: (**A**,**B**) Western blot analysis of Grm1. (**C**) Western blot analysis of Arhgap4. (**D**) Western blot analysis of Ntsr1. (**E**,**F**) Slc39a12 quantification via ELISA. Results presented as median and min–max of (4–9) animals per group. Each dot in the plots = 1 animal. Statistical significance: * *p* < 0.05; ** *p* < 0.01; *** *p* < 0.001. NJ—normobilirubinemic Gunn rats (triangle symbols, white box); LP—low-phenotype hyperbilirubinemic Gunn rats (circle symbols, yellow box); SP—severe-phenotype hyperbilirubinemic Gunn rats (square symbols, orange box); Cll—cerebellum; hCtx—parietal motor cortex; Arhgap4—Rho-GTPase-activating protein 4; Grm1—glutamate metabotropic receptor 1; Ntsr1—neurotensin receptor 1; Slc39a12—solute carrier family 39 member 12.

Western blot analysis showed a phenotype-dependent decrease in Grm1 proteins in both Cll and hCtx (Figure 9A,B). In Cll, Grm1 protein reduction reached 13.55-fold (*p* < 0.001) and 1.96-fold (*p* < 0.01) in SP and LP, respectively, versus the NJ group, and 6.9-fold in SP vs. LP (*p* < 0.001) (Figure 9A). In the hCtx region, there is a trend toward reduction at 1.76-fold for LP and 3.39-fold for SP, with the latter reaching statistical significance vs. NJ (*p* < 0.05) (Figure 9B). Grm1 downregulation, paralleling the worsening of the phenotype, is highly interesting.

Arhgap4 protein levels in the Cll region were not significantly different among NJ, LP, and SP (Figure 9C), but were not detected in the hCtx. The results suggest that Arhgap4 could not fully explain the worsening of the phenotype.

Ntsr1 protein was only detected in the hCtx region (Figure 9D), where its level was similarly increased in both LP and SP, where only SP was statistically significant versus NJ (*p* < 0.01). Because there was no difference between LP and SP, Nstr1 does not explain the severe phenotype.

Slc39a12 protein (ZIP12) levels measured by ELISA were generally higher in hyperbilirubinemic than in normobilirubinemic animals. However, even though Slc39a12 protein levels of SP vs. NJ in the Cll were found to be statistically significant, they showed a trend toward normalization vs. NJ (Figure 9E), so not in line with the worsening of the phenotype. Differently, Slc39a12 in hCtx, despite the large variability, showed an increasing trend paralleling the phenotypes.

Casp6 proteins play a role as apoptotic effector caspases [105] that could be extrapolated from histological data (see Figure 3 and Figure 4) (reviewed in [106]) even if Casp6 proteins are not detected (Figure S2, Supplementary Materials).

The significant modulations of Grm1 and Slc39a12 at the proteomic level also support the biomolecular connection of bilirubin-induced damage with neuro-behavioral/psychiatric disorders that could manifest in youth and adult age [19].

In summary, the only protein that shows no distinct overlap among NJ, LP, and SP with strong agreement with the worsening of the phenotype is Grm1.

### 2.4. Total Serum Bilirubin (TSB) Concentration and Brain Bilirubin (BrB) Content Do Not Fully Explain the Severe Motor Dysfunction

#### 2.4.1. TSB and BrB in Adult Animals

To investigate if a different amount of bilirubin exposure (the challenge) may explain the severe motor phenotype, we quantified bilirubin in both blood (total serum bilirubin: TSB) and the brain (BrB). Serum albumin concentrations were also analyzed since albumin is a binder of bilirubin in blood, avoiding its entry into the brain [107].

**Figure 10:**
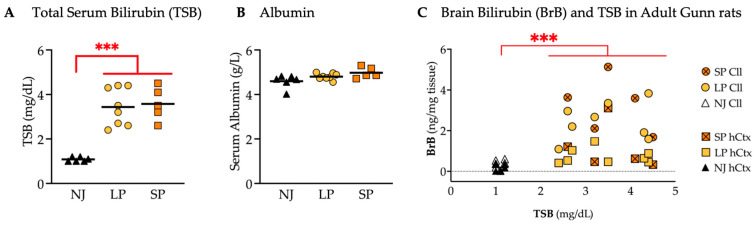
TSB concentrations and bilirubin content in the Cll and hCtx of adult Gunn rats. (**A**) TSB. (**B**) Serum albumin. (**C**) BrB content plotted against TSB. Each dot = 1 animal (5–8 animals per group). Statistical significance: *** *p* < 0.001. NJ—normobilirubinemic Gunn rats (white and black triangles); LP—low-phenotype hyperbilirubinemic Gunn rats (yellow circles and squares); SP—severe-phenotype hyperbilirubinemic Gunn rats (orange circles and orange squares with/without cross); Cll—cerebellum; hCtx—parietal motor cortex.

TSB was significantly higher in jj animals vs. NJ (*p* ≤ 0.001) (Figure 10A), with a good overlap of SP and LP, while overlapping serum albumin concentrations were detected among SP, LP and NJ (Figure 10B).

The amounts of BrB, particularly in the Cll and hCtx regions, were plotted against TSB, each point representing an individual animal (Figure 10C). Hyperbilirubinemic animals (SP and LP) were confirmed to have a significantly higher BrB vs. NJ (*p* ≤ 0.001) but overlapping BrB values between SP and LP were observed in both Cll and hCtx.

These results suggest that the only determinant of BrB is TSB, and that neither TSB nor BrB in these animals (adult) is responsible for the SP.

#### 2.4.2. TSB and BrB from the Early Postnatal Age to Adult Life

**Figure 11:**
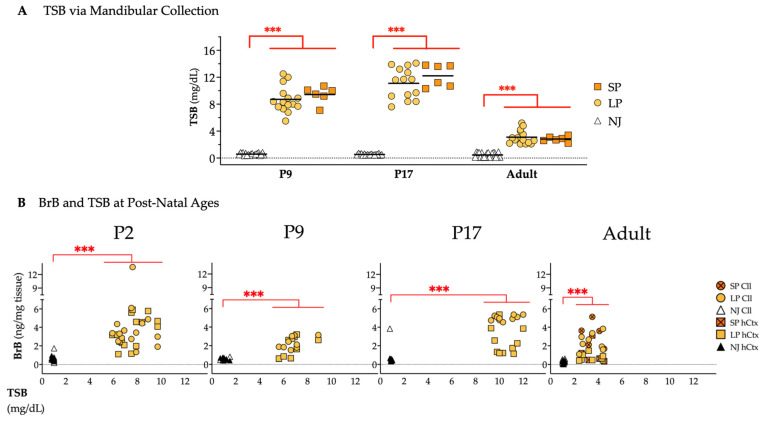
Bilirubin quantity at different postnatal ages. (**A**) TSB from mandibular collection. (**B**) Bilirubin in the Cll and hCtx plotted against TSB in NJ, LP and SP rats. Statistical significance: *** *p* < 0.001. Each dot = 1 animal (6–20 animals per group). NJ—normobilirubinemic Gunn rats (white and black triangles); LP—low-phenotype hyperbilirubinemic Gunn rats (yellow circles and squares); SP—severe-phenotype hyperbilirubinemic Gunn rats (orange circles and orange squares with/without cross); Cll—cerebellum; hCtx—parietal motor cortex.

Because the changes we observed in adult SP animals could be due to earlier postnatal bilirubin challenge, we repeated TSB and BrB measurements from P2 to adulthood. The overlap in TSB between SP and LP in TSB was confirmed during postnatal growth (Figure 11A; 15- to 16-fold in P9 (*p* ≤ 0.001); 21- to 23-fold in P17 (*p* ≤ 0.001) and 6- to 7-fold in adults (*p* ≤ 0.001); all higher serum bilirubin concentrations than NJ).

BrB in Cll and hCtx was again higher in jj than in NJ animals at all postnatal ages (P2, P9, P17, adult, all *p* ≤ 0.001, Figure 11B). As observed in adults (repeated to better appreciate the dynamics of postnatal bilirubin challenge), the BrB quantification in jj rats from P2 to P17 presented a highly overlapped, unique cluster for BrB in the Cll and hCtx regions, indicating that the amount of bilirubin in the brain depends on the TSB concentrations. However, neither TSB nor BrB alone could explain the SP/LP.

## 3. Discussion

KSD incorporates the neurological sequelae of bilirubin neurotoxicity during the neonatal period, with motor disabilities showing a large variability in signs and symptoms [1,17,108,109]. However, the biomolecular reasons for this variability are still largely unexplained. We took advantage of our colony of Gunn rats with spontaneous wide motor disability to investigate the molecular determinants of the ongoing behavioral worsening.

This study demonstrated an increase in the scores of damages from NJ to SP through LP rats, paralleling the worsening of balance, motor coordination and performance. The Cll of adult SP rats exhibited extreme underdevelopment and an increased presence of suffering cells, accompanied by a reduced number of neurons (PCs, granular cells, and NeuN+ cells in the molecular layer, both vs. NJ and LP; Figure 3) generally in agreement with the literature on the toxic effects of bilirubin in models [23,24,110,111,112] and in the clinic [113]. While astrogliosis (the activation of astrocytes leading to release of pro-inflammatory mediators and glutamate in the extracellular matrix) is a well-documented reaction to the bilirubin challenge [64,95,114,115,116,117,118], the observed decrease in GFAP signals in the Cll, indicative of a reduction in the Bergmann glia (BG, chiefly responsible for glutamate uptake [119]), is quite new.

Nevertheless, the suffering and death of astrocytes (LDH release, apoptosis, MTT metabolism, GFAP decrease) together with the inhibition of glutamate uptake have been observed in vitro/ex vivo [117,120,121,122]. This supports the concept that very strong and advanced damage is ongoing in the adult SP animals we analyzed, as previously reported by Sturrock in the Gunn rat in the 1980s [123].

The suffering and death of PCs may contribute to the disarrangement of glial structures since the cytodifferentiation of BG cells proceeds in correlation with migration, dendritogenesis, synaptogenesis, and maturation of PCS [124]. In SP rats, we also counted a reduction in neurons in the molecular layer, namely the basket cells and stellate cells. Based on Yamamura’s description of the Gunn rats, their absence may be due to the well-known underdevelopment of PCs’ dendrites [112]. Altogether, cerebellar neuron and astroglia loss may contribute to motor disorders with abnormal postures, balance and coordination deficits like dystonia and ataxia, thus being in good agreement with the spontaneous phenotype displayed by our SP Gunn rats [125,126] and the clinic [1].

Bilirubin toxicity also manifests in the parietal motor cortex (hCtx) [22], where we observed suffering cells, astrogliosis (Figure 4C,F), and permanent transcriptomic alterations (see later), supporting the presence of damage [19,90,127] despite a similar thickness [19]. This observation showed similarity to a case study performed by Wisnowski and colleagues [7] with an infant having a bilirubin-induced neurologic dysfunction score of 9 out of 9. MRI revealed a distinct abnormal signal in key regions within the dentato-thalamo-cortical pathway, in which both cerebral motor cortex and cerebellum are included.

In addition to the morphometric data, we described a phenotype-dependent permanent alteration in the expression of twelve genes involved in key processes of postnatal brain development (Figure 5 and Figure 6) such as synaptogenesis, synaptic activity, and neuronal circuit establishment; migration, differentiation, and morphogenesis; extracellular matrix formation; repair and plasticity; and bioenergetics (summarized in Table 1).

Moreover, the analysis of the rearrangement of the gene clustering in hyperbilirubinemic subjects (Figure 8, Supplementary Tables S3 and S4) suggests the involvement of potential biological processes, signaling pathways, and even diseases in LP and SP animals. Among the newer ones requiring further devoted studies to determine their real role and impact in KSD, multiple mechanisms are already known to act in bilirubin neurotoxicity, e.g., reactive oxygen species (ROS), AMPK and PI3K-AKT- (FoxO, NF-κB, PKC, ERK) [104,128,129,130], the calcium signaling pathway [99,100,101]; apoptosis [94,131], long-term potentiation/depression [96,97,98], glutamatergic synapses [63], and oxidative phosphorylation [132,133] (see red text in Supplementary Tables S3 and S4).

Differences in bilirubin challenge (amount and duration) are a first clinical and experimental explanation for the variability of manifestations of bilirubin toxicity. However, neither TSB nor BrB in adults may explain the differences at the behavioral, histological, and biomolecular levels among LP and SP. Moreover, TSB and BrB do not show significant differences at different postnatal ages among hyperbilirubinemic subjects, further supporting the idea that the amount of bilirubin alone is not a good predictor of the degree of damage. This contrasts with previous studies using in vitro models (immortalized cells, primary cultures, co-cultures, and organotypic brain cultures), which demonstrated that the damage is proportional to the amount of bilirubin [89,90,91,92,93,94,95]. The discrepancy can be clearly explained by the models and experimental scheme. Clinically, an individual’s susceptibility to bilirubin is supposed to be explained by etiologic co-morbidities and the interaction of multiple gene loci. Up to now, only variants limited in increasing TSB have been identified [134,135].

In addition to laying the foundation of personalized medicine, the effect of genetic variability on the modeling of a disease is well-known in mice but also described in Gunn rats (which have a Wistar genetic background) [136]. As an example, when the j gene was transferred to rats of different genetic backgrounds (Sprague Dawley, ACI/N-j strain, RHA/N-j strain), the incidence of KSD features and mortality drastically varied. This demonstrated that inherited factors, other than deficiency of the specific glucanosyltransferase, modulate susceptibility to bilirubin toxicity [137]. This hypothesis supports the phenomena of our Gunn rat colony inheriting the severe phenotype.

An alternative explanation for the variability of symptoms (KSD) claims a differential sensitivity among the different areas of the brain to bilirubin challenge, largely documented by the literature [7,17,90,115,127,138,139,140,141,142], which is supported again by this study. The most striking evidence is shown in Figure 5 (mRNA modulation) and especially Figure 7 and Figure 8 (dendrogram and “functional clusters”). Each region demonstrates a different reaction to bilirubin challenge in hyperbilirubinemic (jj) rats (LP/SP). Notably, differences among the two regions are already present at physiological level (NJ) and expected to exist because each region is composed of varying populations of specialized neural cells (Purkinje, pyramidal, granular cells; Bergmann, fibrous, protoplasmic, radial glia) in addition to different glia/neuron ratios [143]. This reasonably might explain the different responsiveness. Nevertheless, and again, due to the equal bilirubin challenge among LP and SP since birth to adult age analyzed, physiological differences among regions are not enough to explain the variability of the LP/SP. This opens the possibility that the hypothesized individual genetic variants act in the molecular interplay with bilirubin challenge at the CNS level. Our experimental scheme may not be best suited to identify these responsible variants because we focus on following only 20 markers. More appropriate approaches are ongoing.

Of maximum relevance among all the 20 studied targets, Grm1 is the unique gene of bilirubin neurotoxicity that is consistently downregulated in all the regions of the hyperbilirubinemic rats that we have evaluated up to now (not only hCtx and Cll, but also frontal Ctx, hippocampus, and inferior colliculi, attending to memory, cognition and auditory functions) [19], suggesting a promising candidate marker for ongoing bilirubin-induced damage.

Grm1 encodes metabotropic glutamate receptor (mGluR) 1 proteins, a G-protein-coupled receptor that mediates excitatory neurotransmission in the CNS. It is known that bilirubin inhibits glutamate uptake and increases its concentration in the extracellular matrix by acting on both neurons and astrocytes, enhancing glutamate-mediated Ca^2+^ influx and death, especially in neurons [95,116,121,122,144,145]. Increased glutamate levels have also been reported in in vivo models [21,141], and in kernicterus infants by the use of 1H-MR spectroscopy [141,146]. The literature on the bilirubin field has mainly focused on ionotropic glutamate receptors, reporting conflicting data [64]. Both NMDA and AMPA receptor-mediated neuronal death have been noticed, with selective blockers able to partly (but not fully) reverse it [63,114,120,131,147,148,149]. The ability of bilirubin to inhibit long-term potentiation (LTP) and long-term depression (LTD) in the hippocampus is known, possibly through calpain-mediated proteolytic cleavage of NMDA receptor subunits [64,96]. Other authors found that bilirubin did not modulate the receptor-gated currents generated by NMDA and AMPA in hippocampal pyramidal cells, nor did the glutamate transporter link currents in retinal glial cells [150]. At toxic levels, bilirubin increases protein kinase C (PKC activity, activating NF-κB, responsible for many of the toxic effects of the pigment (inflammation, ER stress, etc.) [151,152,153]) and decreases the expression of NMDA [152,153]. Bilirubin also acts on GABA (gamma-aminobutyric acid—inhibitory, made from glycine/glutamate in the cytoplasm of the presynaptic neuron [154] receptors) by increasing inhibitory postsynaptic currents via protein kinase A (PKA) activation [155,156], in a way that is dependent on the concentration of Ca^2+^ in the synaptic cleft. In agreement, blocking the GABA/glycine receptors attenuates the bilirubin-induced neuronal firing rate [157]. Bilirubin, still via PKA, will also facilitate GABA (and glycine) release in the rat ventral cochlear nucleus (auditory system, with auditory abnormalities representing one of the first signs of bilirubin toxicity to the brain [1,155]). Notably, at the post-synaptic level, multiple glutamate-linked receptors may be co-expressed and strictly cross-linked during brain development [158,159]. Thus, the discrepancies possibly reflect the diversity among the models indicated through analyses on the areas of the brain, intensity of the bilirubin challenge, and developmental stage. As a glutamate metabotropic receptor, Grm1 acts in multiple ways. Extracellular glutamate activates Grm1, inducing the production of inositol triphosphate (IP3) and diacylglycerol (DAG). IP3 works in releasing Ca^2+^ from intracellular storage [160] and in increasing cation (including Ca^2+^) entry into the cell via transient receptor potential canonical proteins (TRPCs) [160,161]. Increased intracellular Ca^2+^ is the basis of the apoptotic and necrotic processes known as glutamate excitotoxicity [144]. DAG will also activate PKC, in turn leading to transcription of multiple genes, including those involved in synaptogenesis and synaptic plasticity [160,162]. The DAG-PKC signaling is potentiated by the increased Ca^2+^ level both of intracellular and extracellular origin [160,163], with the two mechanisms supposed to work independently [164]. The complexity of glutamatergic signaling at the synapses, in their development and function, may add explanation to the contradictory results reported before and substantiate the increased relevance of glutamate in bilirubin toxicity. Evidence connecting bilirubin to Grm1 and the more discussed AMPA, NMDA and GABA transporters exists. Guo et al. [165] reported that bilirubin inhibited Grm1 by activating GABAA (inotropic) and inhibiting GABAB (metabotropic) receptors by finally reducing the PC–granular cell connections involved not only in balance, but also in eye movement, with upward gaze as one classical sign of ABE [1]. By activating PKC [166], bilirubin may indirectly render the neurons sensitive to glutamate toxicity mediated by Grm1. The Grm1–PKC cascade requires binding to DAG and Ca^2+^ is key in PC development; PKC-deficient mice showed increased intracellular Ca^2+^ level and LTD (long-term depression) due to AMPA transporter internalization and ataxia [167]. The ability of the Grm1-IP3 axis to internalize AMPA receptors, triggering LTD, has also been reported [168]. Moreover, acute LTD (in the hippocampus) is induced by the activation of Grm1 in an NMDA-independent but simultaneous way [169]. Although most of the knowledge on glutamate transmission and neurologic dysfunction has been obtained in the cerebellum or hippocampus, we suggest that this knowledge may apply to the entire motor circuits [170,171].

Of translational relevance, most current-era Gun rat colonies need bilirubin albumin displacers to increase the amount of bilirubin entering the brain to manifest signs of ABE/KSD. With the presence of spontaneous SP and LP rats, our actual colony is a strong model of the disease and a powerful tool for studying biomolecular determinants and pre-clinical testing of treatments.

## 4. Materials and Methods

### 4.1. Animals

Gunn rats (Gunn-UGIA1j/BluHsd), with a spontaneous mutation in the UDP glucuronosyl transferase 1a1, the enzyme responsible for bilirubin conjugation and clearance, present hyperbilirubinemia soon after birth, leading to a neurological sequel similar to that in humans [172,173]. Animals at different postnatal ages were obtained from the SPF Animal Facility of the University of Trieste (AREA Science Park, Basovizza). Animals were housed in a temperature-controlled environment (22 ± 2 °C) and on a 12 h light/dark schedule, with ad libitum access to food and water. Animals were sacrificed by decapitation under deep anesthesia (CO_2_ inhalation). The entire litter, composed of both normobilirubinemic (NJ) and hyperbilirubinemic (jj) animals with an evident motor phenotype (SP: tremors, balance problems, difficulty in reaching and maintaining the rear position, dystonia, until wobbly gait), and jj rats with motor deficits detectable only by tests (LP), was included in the study. Based on our experimental experience, which demonstrated that sex is not relevant for this model, both male and female pups were used. A total number of 149 animals were used. In detail, 57 rats were younger than P17 (23 NJ and 34 “jj” hyperbilirubinemic); 92 were adult (30 NJ, 36 LP, and 26 SP). In respect of the 3R rule, when feasible, each animal was used for multiple purposes (e.g., immunofluorescence/histology, qPCR/protein analysis). The study was approved by the animal care and use committee of the University of Trieste (OPBA: Organismo Per il Benessere Animale) and the competent Italian Ministry (1024/2020-PR- and NO2134GAZ20). All the procedures were performed according to the Italian Law (D.Lgs.26/2014) and the European Community Directive (2010/63/EU). A maximal effort was made to minimize the number of animals used and their suffering (3R rule).

### 4.2. Behavioral Tests

To evaluate the potential behavioral abnormalities possibly linked to the altered morphometry/transcriptome data, beam-walking tests were used in adult animals as previously described [19]. Briefly, adult rats (P44 ± 4 days) were placed on a wood beam (width 3 cm × length 100 cm, placed 30 cm from the table’s surface) and allowed to walk independently on the beam. The distance and time of each animal’s performance were recorded and the speed (as a function of centimeter/second) was then calculated. In accordance with the authorization, tests were repeated no more than twice a day, with a recovery time between the two repetitions. Animals were taught to perform the tests by repeating the procedure for three consecutive days. Data were collected on day 3. Data represented at least five animals for each phenotype, and based on the results of a normality test, we applied a parametric, two-tail, unpaired *t*-test.

### 4.3. Cerebellar Weight

Cerebellar weight was recorded with a precision balance immediately after sacrifice and expressed in mg/animal. Data represented at least five animals for each phenotype, and based on the result of the normality test, we applied a parametric, two-tail, unpaired *t*-test.

### 4.4. Histology

Histology was performed at the Pathological Anatomy Unit, Department of Medical, Surgical, and Health Sciences, University of Trieste. Immediately after sacrifice, the brains were collected and fixed in a 4% formalin-buffered solution then embedded in paraffin. Sagittal sections (ML 1.1 to 1.5, stereotaxic coordinate in agreement with the interactive online Paxinos rat brain atlas http://labs.gaidi.ca/rat-brain-atlas/?ml=1.1&ap=-3&dv; accessed on 4 April 2023) of 3–5 μm thickness were obtained by a microtome (RM 2255, Leica Microsystem, Milan, Italy). Hematoxylin and eosin staining was performed by an automated slide stainer Tissue-Tek Prisma Plus (Sakura Finetek Europe B.V.9). Staining was performed as follows: xylol 2 × 5 min; ethanol 2 × 4 min; H_2_O 1 × 3 min; hematoxylin 12 min; H_2_O 2 × 6 min; eosin 1 × 1.30 min; ethanol 2 × 3 min; xylol 1 × 3 min plus 1 × 2 min. Images were collected and analyzed with a D-Sight plus image digital microscope and scanner (Menarini Diagnostics, Firenze, Italy) by two pathologists blinded to the experimental design. The tissue thickness was quantified in the hCtx region, thanks to the presence of recognizable anatomical references (Figure S1). Purkinje and granular cells were counted in at least 3 fields/animal under 10× magnification, and at least four animals for each phenotype as described in Supplementary Materials (Figure S1).

### 4.5. Immunofluorescence Microscopy

To count neurons in the hCtx and the molecular layers of the cerebellum, anti-Neun antibody was used, while anti-GFAP antibody was used to visualize astrocytes and related structures. Briefly, fresh rat brains were embedded in OCT immediately after collection and frozen in −80 °C before being cryosectioned using a cryostat (Bright) at −21 °C. Sagittal brain sections (15 μm thick) were mounted on positively charged glass slides and processed for immunofluorescence staining. Briefly, brain sections were hydrated with 1× PBS at room temperature and fixed using 4% PFA in 0.1 M PBS- at room temperature overnight. Then, the slides were washed three times with 1× PBS before incubating with blocking and permeabilizing buffer (5% BSA 5%, NGS 5%, Triton X-100 0.03% in PBS 1×) for one hour at room temperature. Sections were incubated with primary antibodies overnight at 4 °C. After incubation, the sections were washed with blocking and permeabilizing buffer three times before incubation with a suitable secondary antibody against the primary antibodies for two hours at room temperature. The protein targets and the corresponding dilution of specific primary antibodies used are detailed in Supplementary Materials Table S2. Next, the slices were washed with blocking and permeabilizing buffer and then with PBS 1× before they were counterstained with DAPI (1:10,000, Sigma-Aldrich, St. Louis, MO, USA) at the final concentration of 0.1 μg/mL in PBS 1× (1:10,000). Lastly, sections were washed once with PBS 1× and two times with milliQ water and fixed with immunofluorescence mounting media (Fluorescent Mounting Media, Calbiochem, Germany). Samples were later viewed by using fluorescent microscopy (Leica DM2000) (Leica Microsystems Srl, Solms, Germany). NeuN+ cells in the hCtx were counted in a rectangle crossing the entire length of the structure, identified using the same anatomical references as for measuring its thickness (Figure S1). Data represented three animals for each phenotype.

### 4.6. Gene Expression Analysis

Based on the previous studies that demonstrated significant modulations of genes in hyperbilirubinemic Gunn (jj) rats vs. NJ [19,20,24,88], 20 genes representing relevant neurodevelopmental processes (synaptic plasticity, energy, behavior) were selected. Total RNA was extracted using Eurogold RNA Pure reagent (Euroclone, Milan, Italy) and TRI Reagent (Sigma-Aldrich, Saint Louis, MO, USA] and retrotranscribed with the High-Capacity cDNA Reverse Transcription Kit (Applied Biosystems, Monza, Italy) according to the manufacturer’s instructions in a CFX Duet Real-Time PCR System (Bio-Rad Laboratories, Hercules, CA, USA) at 25 °C for 5 min, 37 °C for 120 min, and 85 °C for 5 min. The expression of the genes was assessed in the parietal cortex (hCtx) and the cerebellum (Cll) of adult Gunn rats by real-time PCR (RT-qPCR), as previously described [19,20,24,88]. Samples were analyzed in a CFX Duet Real-Time PCR System (Bio-Rad Laboratories, Hercules, CA, USA). PCR was performed by mixing 250 nM of each of the gene-specific forward and reverse primer pairs, 25 ng of the cDNA template (except for *Bmp5*, *Cacna2d4*, *Camlg*, *Hyal4*, *Ndufs7*, *Ntsr1*, *Pfkfb1*, *Slc39a12*, and *Tnr*, which needed 5 ng of DNA, and *Ndufb8* and *Ptn* that required 1 ng of cDNA), and 1xSsoAdvance™ SYBR^®^ Green Supermix (Bio-Rad Laboratories, Hercules, CA, USA). The amplification protocol used was as follows: initial denaturation at 95 °C for 30 s, followed by 40 cycles of amplification (denaturation at 95 °C for 5 s, annealing at 60 °C for 20 s, and extension at 72 °C for 30 s), and final extension at 95 °C for 1 min. A different protocol was used for *Cacna2d4*, *Casp6*, *Col4a3*, *Grm1*, *Ntsr1*, *Ptn*, *Slit3*, and *Thbs2*: initial denaturation at 95 °C for 30 s, followed by 40 cycles of amplification (denaturation at 95 °C for 5 s and annealing at 60 °C for 20 s), and final extension at 95 °C for 1 min. Melting curve analysis was always performed at the end of each run to assess and confirm product specificity. The relative quantification was made using CFX Maestro Version 2.3 (Bio-Rad Laboratories, Hercules, CA, USA) via the Pfaffl modification of the ∆∆CT equation, accounting for the amplification efficiencies of individual genes. The results were normalized to the housekeeping gene (*Hprt1*) and the level of mRNA was expressed relative to a reference sample [174,175,176]. The data are representative of at least five animals of each phenotypic group.

### 4.7. Western Blot

For Western blot analysis, 60 μg total protein per sample from Cll and hCtx tissues was resolved in 7.5% or 10% SDS–polyacrylamide gel depending on the target protein size. The resolved proteins in the gel were transferred to polyvinylidene difluoride (PVDF) membranes (0.2 μm; Whatman Schleicher and Schuell, Dassel, Germany) at 500 mA for 1 h. The membranes were then blocked with either 4% milk (for actin and Grm1 protein detection) or 5% BSA (for Arhgap4 and Ntsr1 protein detection) in 1× T-TBS (0.2% Tween 20, 20 mM Tris-HCl pH 7.5, 500 mM NaCl) and incubated with specific primary antibodies overnight at 4 °C. Membranes were washed with blocking solution thrice and then incubated with suitable dilutions of HRP-conjugated secondary antibodies (anti-rabbit antibody for anti-actin, anti-Grm1 protein, and anti-Arhgap4 primary antibodies; anti-mouse antibody for anti-Ntsr1 primary antibody) for at least 2 h at room temperature. The membranes were washed once each with blocking solution, 1× T-TBS, and 1× TBS. The list of antibodies is listed in Supplementary Materials Table S2. The band signal was developed via chemiluminescence (Immobilon^®^ Classico Western HRP Substrate, EMD Millipore, Burlington, MA, USA) and visualized using either C-DiGit^®^ Blot Scanner (Li-Cor, LI-COR GmbH, Bad Homburg, Germany), X-ray films (Kodak^®^ BioMax™ Light Film, Rochester, NY, USA), or both. The intensity of the band of both the target protein and actin was quantified by ImageJ software version 1.54g (US NIH, Bethesda, Maryland [Rasband, W.S., ImageJ, U.S. National Institutes of Health, Bethesda, MD, USA, https://imagej.net/ij/ accessed, downloaded, and installed on computer last 5 September 2024). The area of the target band was divided by the area of the actin signal of each sample lane, allowing us to prevent bias due to different loads (normalization). The expression of the targets in SP and LP rats was expressed as a fold change compared to the level of the corresponding target in NJ, serving as the reference (set to 1) in each gel.

### 4.8. ZIP12 (SLC39A12) ELISA Detection

We initially tried to detect Slc39a12 proteins by Western blot analysis. However, only a band between 130 kDa and 180 kDa was observed instead of the target size of 76 kDa. As an alternative, Slc39a12 protein (ZIP12) levels were measured via a Rat Zinc Transporter ZIP12 (SLC39A12) ELISA kit following the manufacturer’s protocol. Briefly, samples were mechanically homogenized using potter tubes and the total protein concentrations were measured by BCA assay. Tissue-specific amounts of proteins were loaded per well to normalize all samples: 350 μg for Cll and 250 μg for hCtx. Standard samples are provided in the kit ready for use. HRP-conjugated reagent was added, 100 μL per well except for the blank well, and incubated at 37 °C for 60 min. Wells were washed four times with 1× wash solution. Then 50 μL of Chrom A solution and 50 μL of Chrom B solution were added per well and incubated for 15 min at 37 °C, protected from light. Stop Solution was added, 50 μL per well, and incubated for 5 min. OD at 450 nm was measured five minutes after adding the Stop Solution. Standard curves were generated by best-fitting curve per batch of runs and used to interpolate the concentrations of Slc39a12 proteins.

### 4.9. Bilirubin Quantification

#### Total Serum Bilirubin

The blood was collected in two ways, depending on the experimental goal. For the determination of the total serum bilirubin level (TSB, mg/dL) in living animals during postnatal growth, animals were unambiguously identified, and the blood was collected by sub-mandibular puncture at P9 and P17 (P, postnatal age in days) after EMLA cream application (lidocaine+ prilocaine). The animals were then left to grow until identifying the phenotype (NJ, LP or SP); thus, the data on TSB at P9 and P17 were assigned to the correct phenotypic subgroup. For this procedure, TSB was not tested at P2, due to the limited blood volume at this age, hindering the possibility of a safe collection. Data are representative of at least 5 animals, and based on the result of a normality test, we applied a parametric, two-tail, unpaired *t*-test.

For animals devoted to brain collection for quantification of bilirubin in Cll and hCtx, blood was collected during the sacrifice.

After collection, blood was left to coagulate at room temperature for 30 min then centrifuged at 2000× *g* for 20 min at room temperature. The serum was transferred to a new tube and stored at −20 °C in the dark until use. TSB was quantified by the diazo reaction [177,178]. Albumin concentration in serum (g/dL) was determined by the bromocresol green method [179]. The absorbance was read at 460 nm.

### 4.10. Brain Bilirubin Measurement

Brain bilirubin concentrations were measured from the Cll and the hCtx following the LC-MS/MS method for the quantitative determination of Z-lumirubin [180]. Briefly, different concentrations (10, 30, 50, 100 and 400 μmol/L) of unconjugated bilirubin (UCB) were prepared to generate the calibration curve by diluting UCB stock solution with methanol containing 0.3% BHT, 0.1% ascorbic acid, and 0.5% ammonium acetate (methanol with antioxidant and ammonium acetate) to prevent degradation of UCB and precipitate residual proteins. The internal standard (ISTD) was prepared by dissolving mesobilirubin (MBR, Frontier Scientific, Logan, UT, USA) in DMSO immediately before use. A homogenate of rat brain tissue was prepared by weighing 2–100 mg of brain tissue and then diluting to the appropriate amount of matrix with a buffer solution (1 mmol/L K_3_PO_4_·H_2_O, pH 7.4). Suspension was homogenized by an ultrasonic homogenizer.

Calibration samples were prepared by adding 10 μL of normobilirubinemic brain homogenate, 10 μL calibration solution of UCB and 10 μL of ISTD (20 µmol/L). Deproteinization was performed after vortex mixing of the samples with 570 μL methanol containing antioxidants and ammonium acetate, followed by centrifugation at 15,000× *g* for 20 min. Then, 150 μL of the final supernatant was transferred to glass vial inserts and 5 μL of the solution was injected into an LC-MS/MS platform. Blank samples were prepared by adding 10 μL of DMSO to 10 μL of brain homogenate and 10 μL of ISTD to measure and subtract the background signal.

Rat brain samples were homogenized in the same manner as for calibration. For LC-MS/MS, 10 μL samples of homogenized rat brain were mixed with 10 μL of ISTD (20 µmol/L). The deproteinization and LC-MS/MS measurement were performed in the same manner as for calibration purposes. The final brain bilirubin content is expressed in ng/mg of tissue.

Because the frequency of SP rats is approximately 30% of total jj, we expected to have at least 3–4 SP rats from P2 to P17.

### 4.11. Statistical Analyses

Data were analyzed using GraphPad InStat for Windows (GraphPad Software 3.1, Inc., La Jolla, CA, USA). The Shapiro–Wilk normality test was used to study the data distribution. Parametric, two-tailed, unpaired, *t*-tests were used for data passing the normality test, while a non-parametric test was used for those data failing the normality check. The only discriminant for failing the normality test was a sample number of 3 or 4. For all, statistical significance was indicated as follows: * *p* < 0.05; ** *p* < 0.01; *** *p* < 0.001.

### 4.12. Correlation Analyses

All correlation analyses were conducted using RStudio version 2024.12.0+467 [181]. Normality tests were first performed between beam walking and gene expression using the Shapiro–Wilk test. The results from the normality test concluded that the Spearman correlation was to be used. Scatterplots were generated using ggplot2 with the ggscatter command, which included a confidence interval, a correlation coefficient (R), and a linear regression line.

The Cll and hCtx data were separated based on phenotype. Gene expression data was grouped for normalization through computing log values to minimize skewness. The preparation of the dendrograms used Agglomerative Hierarchical Clustering (AGNES), which clusters data points for iterative merging based on similarity until it merges into a single cluster [182]. The clustering method used was the complete or maximum linkage, which computed all the pairwise dissimilarities between the gene clusters. This method considers the largest value of dissimilarities as the distance between different clusters.

Gene networks were constructed per phenotype for both Cll and hCtx. These networks were generated based on a linear association process and simulated network prediction based on expression data. Through Pearson’s correlation, a matrix was developed between the genes in preparation for the construction of the adjacency matrix with a similarity threshold of 0.7. Clustering was calculated using the Louvain method, which detects structures by maximizing the strength of network division into communities and measuring the density within values. The plots were then generated with igraph.

## 5. Conclusions

We substantiated that the amount and duration of the bilirubin challenge cannot fully explain the tissue, transcriptomic, proteomic, and neurobehavioral alterations per se, supporting the clinical hypothesis on the contribution of other, possibly genetic factors. Moreover, while the increasing severity of bilirubin-induced dysfunctions does not necessarily correlate with observable structural changes in the CNS, nor does it fully fit with classic MRI analysis (reviewed in [7],) our data support the role of Grm1 and glutamate as immediately translatable markers of bilirubin neurotoxicity. Due to the great agreement with the clinical data, we suggest performing 1H-MR spectroscopy [141,146] in infants with suspicion of ongoing bilirubin neurologic damage, which might improve diagnosis and management, and avoid cases of KSD.

## Figures and Tables

**Table 1:** Details on the biological functions of the studied genes based on the literature.

Biological Process	Gene	Short Description	References
Migration, Differentiation, Morphogenesis	*Slit3*	Acts as chemo-repellents in axonal guidance	[25]
	*Tnr*	Neuron and neurite growth, synapse maintenance, oligodendrocyte differentiation, regulates astrocyte glutamate uptake in adult brain	[25,26,27,28]
	*Casp6*	Apoptosis, important for achieving the final architecture of the mature, functional central nervous system (CNS).	[19,24,29,30,31,32]
	*Thbs2*	Presynaptic formation during development, cell–cell and cell–matrix interactions; compartmentalization of the extracellular matrix	[25,33,34,35]
	*Arhgap4*	Neurogenesis, synaptogenesis, development, inhibition of cell motility and axon outgrowth and repair during brain development and synaptic plasticity by inhibiting cell and axon motility and regulation of actin.	[24,36,37,38,39,40,41,42,43]
Extracellular Matrix (ECM)	*Col4a3*	Neuronal development and overall structure of the brain	[25,44,45]
	*Hyal4*	Extracellular matrix component, limits lateral diffusion of AMPA receptors; promotes activity of L-type Ca^2+^ channels	[25,46,47]
Synaptogenesis, Synaptic activity, Neuronal circuits establishment	*Thbs2*	Presynaptic formation during development, cell–cell and cell–matrix interactions; compartmentalization of the extracellular matrix	[25,33,34,35]
	*Cacna2d4*	Voltage-gated calcium channel, with the α2δ subunits as important regulators of synapse formation	[27,48]
	*Bmp5*	Extension and survival of dendrites	[46,47,49]
	*Grm1*	Glutamate neurotoxicity, intracellular signals via interactions with G proteins, and synaptic activity. Glutamate is one of the major neurotransmitters in the brain and is relevant to brain development, with glutamate neurotoxicity considered one of the principal mechanisms of bilirubin neurotoxicity; it has been involved in autism, schizophrenia, and bipolar disorders.	[19,27,48,50,51,52,53,54,55,56,57,58,59,60,61,62,63,64,65]
	*Ntsr1*	G-protein-coupled receptors relevant to synaptogenesis, plasticity, and neuronal circuity. Neurotensin that acts on its receptor is involved in increased synaptic excitability in different mesencephalic and dopamine cortical neurons via glutamate outflow enhancement and glutamate receptor activation, which subsequently amplifies glutamate-induced neurotoxicity	[27,50,55,58,59,61,65,66,67,68,69,70,71,72,73,74,75]
	*Camlg*	Membrane trafficking of postsynaptic GABAA receptors	[48,60]
	*Slit3*	Acts as chemo-repellent in axonal guidance	[25]
	*Arhgap4*	Neurogenesis, synaptogenesis, development, inhibition of cell motility and axon outgrowth and repair during brain development and synaptic plasticity by inhibiting cell and axon motility and regulation of actin.	[24,36,37,38,39,40,41,42,43]
Repair Plasticity	*Bmp5*	Extension and survival of dendrites	[46,47,49]
	*Ntsr1*	G-protein-coupled receptors relevant to synaptogenesis, plasticity, and neuronal circuity. Neurotensin that acts on its receptor is involved in increased synaptic excitability in different mesencephalic and dopamine cortical neurons via glutamate outflow enhancement and glutamate receptor activation, which subsequently amplifies glutamate-induced neurotoxicity	[27,50,55,58,59,61,65,66,67,68,69,70,71,72,73,74,75]
	*Ptn*	Repair and plasticity	[76,77,78,79]
Energy	*Ndufs7/8*	Important subunit of mitochondrial respiratory chain crucial for atp production	[27,80,81,82]
	*Pfkfb1*	Catalyzes both the synthesis (glycolysis) and degradation (gluconeogenesis) of fructose-2,6-biphosphate.	[83,84]
Behavior	*Camlg*	Membrane trafficking of postsynaptic GABAA receptors	[48,60]
	*Grm1*	Glutamate neurotoxicity, intracellular signals via interactions with G proteins, and synaptic activity. Glutamate is one of the major neurotransmitters in the brain and is relevant to brain development, with glutamate neurotoxicity considered one of the principal mechanisms of bilirubin neurotoxicity; it has been involved in autism, schizophrenia, and bipolar disorders.	[19,27,48,50,51,52,53,54,55,56,57,58,59,60,61,62,63,64,65]
	*Slc39a12*	Zn transporter involved in control of gene transcription, growth, development, and differentiation, postsynaptic and neuronal circuit functions. Increased ZIP12 would increase cytoplasmic Zn^2+^ in the extracellular space and intracellular compartments and induce zinc neurotoxicity. It has been involved in schizophrenia.	[85,86,87]
	*Cacna2d4*	Voltage-gated calcium channel, with the α2δ subunits as important regulators of synapse formation	[27,48]
	*Tnr*	Neuron and neurite growth, synapse maintenance, oligodendrocyte differentiation, regulates astrocyte glutamate uptake in adult brain	[25,26,27,28]
Bilirubin oxidation	*Cyp1a1*, *1a2*, *2a3*	Bilirubin oxidation	[20,88]

*Arhgap4*—Rho-GTPase-activating protein 4; *Bmp5*—bone morphogenetic protein 5; *Cacna2d4*—calcium voltage-dependent calcium channel complex alpha-2/delta subunit family; *Camlg*—calcium modulating ligand; *Casp6*—caspase 6; *Col4a3*—collagenase 4a3; *Cyp1a1/1a2/2a3*—cytochrome P450 1a1/1a2/2a3; *Grm1*—glutamate metabotropic receptor 1; *Hyal4*—hyaluronic acid 4; *Ndufs7/b8*: NADH—ubiquinone oxidoreductase (complex I) subunit 7/8; *Ntsr1*—neurotensin receptor 1; *Pfkfb1*—6-phosphofructo-2-kinase/fructose-2,6-biphosphatase 1; *Ptn*—pleiotrophin; *Slc39a12*—solute carrier family 39 member 12; *Slit3*—slit guidance ligand 3; *Thbs2*—thrombospondin 2; *Tnr*—tenascin R.

## Data Availability

All the data related to this study are already available in the paper or in the Supplementary Materials.

## Supplementary Materials

The following supporting information can be downloaded at https://www.mdpi.com/article/10.3390/ijms26136262/s1.

## Abbreviations

The following abbreviations are used in this manuscript:

ABE	Acute Bilirubin Encephalopathy
AKT	Protein kinase B
AMPA	α-Ammino-3-idrossi-5-Metil-4-isossazol-Propionic Acid
AMPK	Adenosine Monophosphate-activated protein Kinase
Arhgap4	Rho GTPase-activating protein 4
BG	Bergmann glia
Bmp5	Bone morphogenetic protein 5
BrB	Brain bilirubin
Cacna2d4	Calcium voltage-dependent calcium channel complex alpha-2/delta subunit family
Camlg	Calcium modulating ligand
Casp6	Caspase 6
Cll	Cerebellum
CNS	Central Nervous System
Col4a3	Collagenase 4a3
CYP	Cytochrome P450
Cyp1a1	Cytochrome P450 1a1
Cyp1a2	Cytochrome P450 1a2
Cyp2a3	Cytochrome P450 2a3
CP	Cerebral palsy
DAG	Diacylglycerol
ECM	Extracellular matrix
ERK	Extracellular signal-Regulated Kinase
fCtx	Frontal cortex
FoxO	Forkhead box O
GABA	Gamma-AminoButyric Acid
GFAP	Glial Fibrillary Acid Protein
GL	Granular Layer
Grm1	Glutamatergic metabotropic receptor 1
hCtx	Parietal cortex
Hyal4	Hyaluronic acid 4
IP3	Inositol-1,4,5-triphosphate
ISTD	Internal standard
jj	Hyperbilirubinemic
KSD	Kernicterus Spectrum Disorder
LDH	Lactate dehydrogenase
LTD	Long-Term Depression
LTP	Long-Term Potentiation
LP	Low Phenotype
MBR	Mesobilirubin
ML	Molecular Layer
MRI	Magnetic Resonance Imaging
Ndufb8	NADH-ubiquinone oxidoreductase complex 1 subunit 8
Ndufs7	NADH-ubiquinone oxidoreductase complex 1 subunit 7
NeuN	Neuronal Nuclear Antigen and Neuron Differentiation Marker
NF-κB	Nuclear factor kappa B
NJ	Normobilirubinemic
NMDA	N-methyl-D-aspartic acid
Ntsr1	Neurotensin receptor 1
PCs	Purkinje cells
Pfkfb1	6-phosphofructo-2-kinase/fructose-2,6-biphosphatase 1
PI3K	Phosphatidylinositol 3-kinase
PKA	Protein Kinase A
PKC	Protein Kinase C
Ptn	Pleiotrophin
P2	Postnatal day 2
P17	Postnatal day 17
ROS	Reactive Oxygen Species
Slc39a12	Solute carrier family 39 member 12
Slit3	Slit guidance ligand 3
SPF	Specific-Pathogen-Free
SP	Severe Phenotype
Thbs2	Thrombospondin 2
Tnr	Tenascin r
TRPCs	Transient Receptor Potential Canonical proteins
TSB	Total Serum Bilirubin
UCB	Unconjugated bilirubin
UDPGT	UDP glucuronyltransferase
UGT1A1	Uridine diphosphate-glucuronosyltransferase 1A1
