# Supplementary material for: Transcriptomic and Proteomic Changes in the Brain Along with Increasing Phenotypic Severity in a Rat Model of Neonatal Hyperbilirubinemia
Source: Int J Mol Sci. 2025 Jun 28;26(13):6262. doi: 10.3390/ijms26136262 (PMC12249923; doi:10.3390/ijms26136262)
Supplement: Supplementary file 1 [file ijms-26-06262-s001.zip › ijms-3658584-supplementary.pdf]

Supplementary Materials

Figure S1

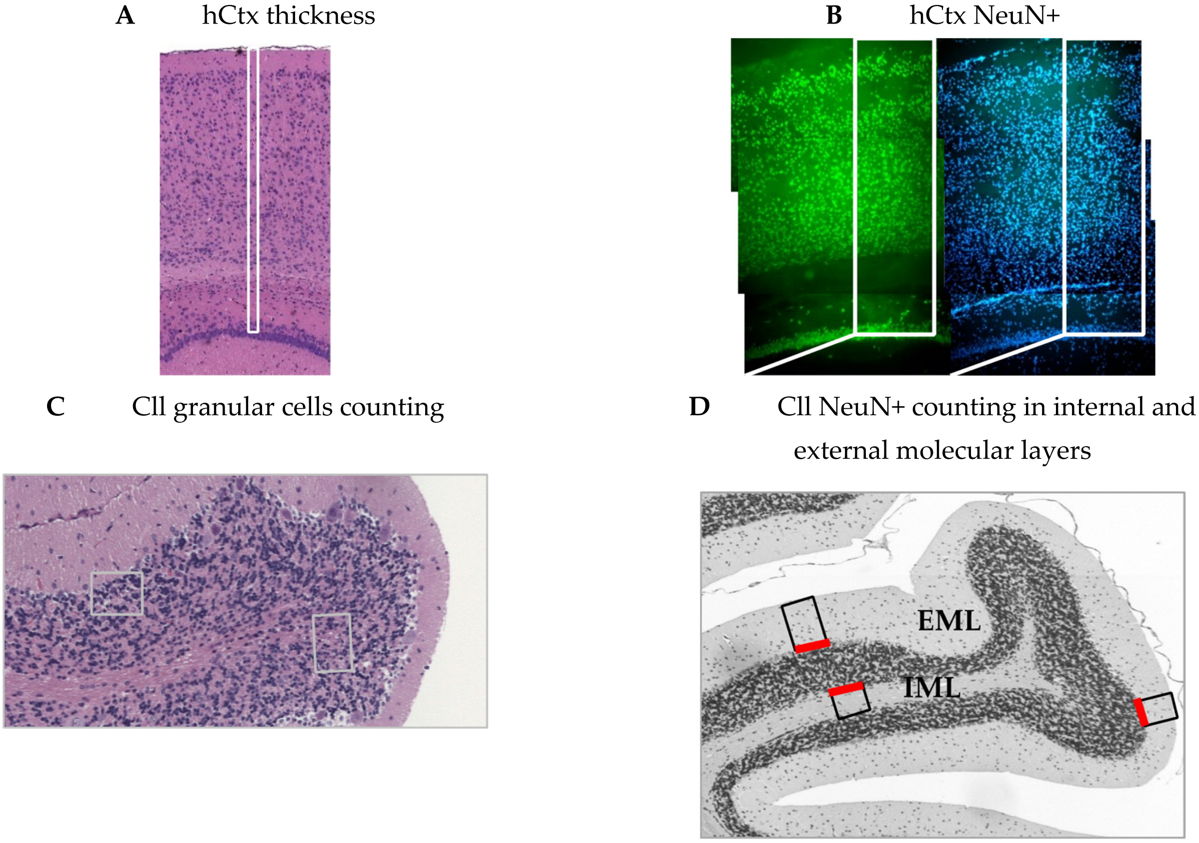

Figure S1: Representative pictures of the fields used for quantifyimg hCtx thicness, NeuN+ cells in hCtx, granular and NeuN+ cells in Cll. 1) The thickness of hCtx has been measured from the boundary between hippocampus and hCtx, to the end of hCtx. 2) Again using hippocampus as reference point, a rectangle of identical dimensions among NJ, LP and SP has been drawn, and all positive (green) cells inside has been counted. Due to the extreme difference in the dimensions of NJ, LP and SP cebella, we decided to count neurons as follow. 3) For counting granular cells we identified a rectangle appropriated to the SP Cll, and replicate it identically in the other phenotypes. At least 3 fields per animal were considered. 4) for NeuN+ neurons in the internal and external molecular layer, we set up a rectangle with a base of a fixed size (red). The height of the rectangle went from a) the granular layer to the end of the tissue (external molecular layer, EML); b) from one granular layer to the opposite one (internal molecular layer, IML). At leas 3 reactable each layer, each animal have been counted. hCtx: parietal cortex; Cll: cerebellum.

Paper 2 Supplementary Materials

Table S1. Amplification efficiencies and sequences of forward and reverse primers of target genes used in the study.

Gene	Final cDNA (ng)	Efficiency (%)	Forward Primer Sequence (5′–3′)	Reverse Primer Sequence (5′–3′)
Hprt1	25	94.9	AGACTGAAGAGCTACTGTAATGAC	GGCTGTACTGCTTGACCAAG
Arhgap4	25	88.0	CTTGTGAGCCATCTACTATC	GTTGAGGAAGGTGAAGAG
Bmp5	5	70.5	CAGTCTACACGATACCAAT	GTAATGCCTTCTCTGATGA
Cacna2d4	5	69.5	CGTCTATATGTCCGAACG	AATACTGCCAGGTCAATG
Camlg	5	70.3	TTGTCTATATTCGCTCCATT	CACTGTCGTCTTTACCTT
Casp6	25	102.2	ACAGATGGCTTCTACAGA	AGTTCCTCTCCTCTTGTG
Col4a3	25	94.5	TCACCACAATGCCATTCTTA	CGACAGCCAGTATGAATAGT
Cyp1a1	25	91.5	CAGGCGAGAAGGTGGATATGAC	GGTCTGTGTTTCTGACTGAAGTTG
Cyp1a2	25	116.9	GTGGTGGAATCGGTGGCTAATGTC	GGGCTGGGTTGGGCAGGTAG
Cyp2a3	25	109.2	ACACAGGCACCCCAGGACATC	ACACAGGCACCCCAGGACATC
Grm1	25	93.3	TATATCATTCTGGCTGGTATT	AGGATGTGGTAGTAGGTT
Hyal4	5	98.4	ACCCATCAATGGTGGTCTTC	GCGCCAATATTCCCAGTCTA
Ndufb8	1	86.9	CAAGAAGTATAATATGCGTGTG	ATCGGGTAGTCACCATAC
Ndufs7	5	88.4	GCTACTACCACTACTCCTACT	CAGCCTGGCACATAGATG
Ntsr1	5	99.5	TCGGATGAACAGTGGACTA	GTAGAAGAGAGCGTTGGTTAG
Pfkfb1	5	65.5	CACGCTATCTCAACTGGAT	CTGTAACTCACTGCCTCTC
Ptn	1	58.1	TGAAGACTCAGAGATGTAAGA	AAGCCTGGAACTGGTATT
Slc39a12	5	83.1	CTCTCCTCCTCCTCTATTAC	CTCATATTCTCTGTTGCTCAT
Slit3	25	76.7	TACGCCTAGAACAGAACT	TCTTGCTGATGTCTATTCG
Thbs2	25	78.3	TGATAACAATGAGGACATAGATG	CTGGTTGGAGTTGGAGAT
Tnr	5	86.9	TCCAACTACCAAGACTACC	TTCATTACCGCAGATATTCC

Cll – cerebellum; hCtx—parietal motor cortex; *Arhgap4* – Rho-GTPase activating protein 4; *Bmp5*— bone morphogenetic protein 5; *Cacna2d4*—calcium voltage-dependent calcium channel complex alpha-2/delta subunit family; *Camlg*— calcium modulating ligand; *Casp6*—caspase 6; *Col4a3*—collagenase 4a3; *Cyp1a1/1a2/2a3/2a5* —cytochrome P450 1a1/1a2/2a3/2a5 (*Cyp2a3* in rats = *Cyp2a5* in humans); *Grm1*—glutamate metabotropic receptor 1; *Hyal4*—hyaluronic acid 4; *Ndufs7/b8* —NADH—ubiquinone oxidoreductase (complex I) subunit 7/8; *Ntsr1*—neurotensin receptor 1; *Pfkfb1*—6-phosphofructo-2-kinase/fructose- 2,6-biphosphatase 1; *Ptn*—pleiotrophin; *Slc39a12*—solute carrier family 39 member 12; *Slit3*—slit guidance ligand 3; *Thbs2*—thrombospondin 2; *Tnr*—tenascin R.

Paper 2 Supplementary Materials

Figure S2

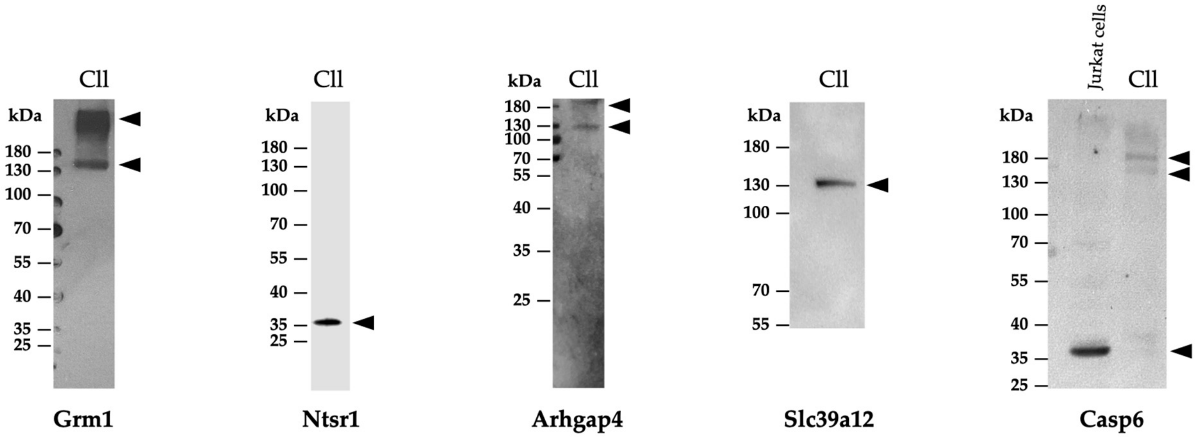

Figure S2. Immunoblots of antibody validation. Grm1 proteins have expected bands at 132-145 kDa and >300kDa (monomeric and dimeric forms, respectively) which are detected. Single band of proteins were detected in Ntsr1. Arhgap4 proteins are expected to have a single band between 100-130kDa, so the lower band is only used for quantification. Slc39a12 proteins have an expected singe band at 70-76kDa, but only a band size at ~130kDa; Slc39a12 (ZIP12) ELISA kit was used to quantify the ZIP12 proteins. Anti-Casp6 antibody detected in at the expected protein size in recommended controls but not in the brain samples. Cll – cerebellum; *Arhgap4* – Rho-GTPase activating protein 4; *Casp6*—caspase 6; *Grm1*—glutamate metabotropic receptor 1; *Ntsr1*—neurotensin receptor 1; *Slc39a12*—solute carrier family 39 member 12; *Thbs2*—thrombospondin 2.

Paper 2 Supplementary Materials

Table S2. Dilution and antibodies used for protein detection using Western blot and immunofluorescence staining.

**Gene-encoded Protein and Expected Size**	**Primary Antibody (Ref.No. and Source)**	**Working Dilution**	**Secondary Antibody (Dilution = 1:1000 to 1:2000)**
**Actin** (42kDa)	Rabbit Anti-Actins Antibody (A2066, Sigma-Aldrich)	WB = 1:2000 (for both)	Polyclonal Goat Anti-Rabbit Immunoglobulins/HRP (P0448, Dako/Agilent)
**Grm1** (132-145 kDa and >300kDa)	Rabbit Anti-mGluR1 mAb (#12551, Cell Signaling & Technology) Lot No. 2 = [512 mcg/mL]	Stock Concentration = 512 mcg/mL WB = 1:1000 (for Cll) WB = 1:100 (for hCtx)	Polyclonal Goat Anti-Rabbit Immunoglobulins/HRP (P0448, Dako/Agilent)
**Arhgap4** (between 100kDa and 130kDa)	Rabbit Anti-ARHGAP4 pAb (HPA001012, Sigma-Aldrich) Lot No. A82750 = [200 mcg/mL]	Stock Concentration = 200 mcg/mL WB = 1:100 (for both)	Polyclonal Goat Anti-Rabbit Immunoglobulins/HRP (P0448, Dako/Agilent)
**Slc39a12** (70-76kDa)	Rabbit Anti-SLC39A12 pAb (NBP2-75665, NovusBio)	WB = 1:100 (for both)	Polyclonal Goat Anti-Rabbit Immunoglobulins/HRP (P0448, Dako/Agilent)
**Ntsr1** (52-54kDa)	Mouse Anti-NTR1 mAb (sc-374492, Santa Cruz Biotech) , Lot Nos: D1613; J1411 [200 mcg/mL]	Stock Concentration = 200 mcg/mL WB = 1:100 (for both)	Polyclonal Rabbit Anti-Mouse Immunoglobulins/HRP (P0260, Dako/Agilent)
**NeuN**	Mouse Anti-NeuN mAb clone A60 (MAB377, Sigma-Aldrich)	IF = 1:2000	Donkey Anti-Mouse Alexa Fluor(TM) 488 (A21202) 1:500
**GFAP**	Rabbit Anti-Glial Fibrillary Acidic Protein (GFAP) pAb (Z30334, Dako/Agilent)	IF = 1:600	Goat Anti-Rabbit Alexa Fluor(TM) 488 (A11008, Invitrogen) 1:1000
WB – western blot; IF – immunofluorescence; mAb – monoclonal antibody; pAb – polyclonal antibody; *Arhgap4* – Rho-GTPase activating protein 4; *Casp6*—caspase 6; *Grm1*—glutamate metabotropic receptor 1; *Ntsr1*—neurotensin receptor 1; *Slc39a12*—solute carrier family 39 member 12; *Thbs2*—thrombospondin 2.			

Paper 2 Supplementary Materials

Table S3 Cll

**Cll NJ**		
Cluster	**Genes**	**Function, signaling pathway, disease**
1	*Arhgap4, Ntsr1, Pfkfb1*	Fructose and mannose metabolism, Glucagon signaling pathway, AMPK signaling pathway (S. Zhang et al., 2022; Y. Zhang et al., 2024)
2	*Cyp1a1, Cyp1a2, Cyp2a3, Ndufb8, Slc39a12*	Retinol metabolism, Chemical carcinogenesis - reactive oxygen species (Lambert et al., 2023; Llido et al., 2023; Thomas et al., 2022; Wagner et al., 2021), Tryptophan metabolism, Metabolism of xenobiotics by cytochrome P450 (Johnson et al., 2012), Chemical carcinogenesis - DNA adducts (Asad et al., 2002), Steroid hormone biosynthesis
3	*Grm1, Thbs2*	not statistically significant output
4	*Casp6, Col4a3, Ndufs7, Camlg, Cacna2d4, Ptn*	not statistically significant output
**alone**	*Bmp5*	TGF-beta signaling pathway, Hippo signaling pathway, Cytokine-cytokine receptor interaction
	Hyal4	Glycosaminoglycan degradation, Lysosome
	Slit3	not statistically significant output
	Tnr	ECM-receptor interaction, Focal adhesion, MicroRNAs in cancer, PI3K-Akt signaling pathway (Ikeda et al., 2015), Human papillomavirus infection
**Cll LP**		
**Cluster**	**Genes**	**Function, signaling pathway, disease**
**5**	*Slit3, Col4a3, Cyp1a1, Hyal4*	not statistically significant output
**6**	*Cacna2d4, Thbs2*	Malaria, Arrhythmogenic right ventricular cardiomyopathy, ECM-receptor interaction, Cardiac muscle contraction, Hypertrophic cardiomyopathy, Dilated cardiomyopathy
**7**	*Ndufs7, Slc39a12*	Parkinson disease, Alzheimer disease
**alone**	*Arhgap4*	Fructose and mannose metabolism, Glucagon signaling pathway, AMPK signaling pathway (S. Zhang et al., 2022; Y. Zhang et al., 2024)
	Bmp5	TGF-beta signaling pathway, Hippo signaling pathway, Cytokine-cytokine receptor interaction
	Camlg	Retinol metabolism, Chemical carcinogenesis - reactive oxygen species (Lambert et al., 2023; Llido et al., 2023; Thomas et al., 2022; Wagner et al., 2021), Tryptophan

		metabolism, Metabolism of xenobiotics by cytochrome P450 (Johnson et al., 2012), Chemical carcinogenesis - DNA adducts (Asad et al., 2002), Steroid hormone biosynthesis
	Casp6	Apoptosis (Grojean et al., 2000; Rodrigues et al., 2002), Lipid and atherosclerosis
	Cyp1a2	Linoleic acid metabolism, Tryptophan metabolism Drug metabolism - cytochrome P450 (M. Zhang et al., 2024), Metabolism of xenobiotics by cytochrome P450 (Johnson et al., 2012), Chemical carcinogenesis - DNA adducts (Asad et al., 2002), Steroid hormone biosynthesis, Retinol metabolism, Chemical carcinogenesis - receptor activation, Chemical carcinogenesis - reactive oxygen species (Lambert et al., 2023; Llido et al., 2023; Thomas et al., 2022; Wagner et al., 2021)
	Cyp2a3	Retinol metabolism
	Grm1	Long-term depression, Long-term potentiation (Yang et al., 2019, p. 20; L. Zhang et al., 2003), Gap junction, Taste transduction, Glutamatergic synapse (McDonald et al., 1998), FoxO signaling pathway, Estrogen signaling pathway, Phospholipase D signaling pathway, Spinocerebellar ataxia, Retrograde endocannabinoid signaling
	Ndufb8	Retrograde endocannabinoid signaling, Oxidative phosphorylation (Hansen, 2000; Zetterström & Ernster, 1956), Non-alcoholic fatty liver disease (Ramírez-Mejía et al., 2024; Tian et al., 2016), Diabetic cardiomyopathy, Chemical carcinogenesis - reactive oxygen species (Lambert et al., 2023; Llido et al., 2023; Thomas et al., 2022; Wagner et al., 2021), Thermogenesis, Parkinson disease, Prion disease, Huntington disease, Amyotrophic lateral sclerosis.
	Ntsr1	Calcium signaling pathway (Liang et al., 2017; Rauti et al., 2020; H.-B. Shi et al., 2006), Neuroactive ligand-receptor interaction
	Pfkbk1	Fructose and mannose metabolism, Glucagon signaling pathway, AMPK signaling pathway (S. Zhang et al., 2022; Y. Zhang et al., 2024)
	Ptn	not statistically significant output
	Tnr	ECM-receptor interaction, Focal adhesion, MicroRNAs in cancer, PI3K-Akt signaling pathway (Ikeda et al., 2015), Human papillomavirus infection
**Cll SP**		
**Cluster**	**Genes**	**Function, signaling pathway, disease**
**8**	*Thbs2, Bmp5, Casp6, Col4a3, Cyp1a2, Cyp2a3, Ptn*	ECM-receptor interaction, Retinol metabolism, Focal adhesion, Cytoskeleton in muscle cells
**9**	*Arhgap4, Cacan2d4, Camlg, Cyp1a1, Hyal4,*	not statistically significant output

	Nduf8, Slc39a12, Slit3	
**10**	*Ntsr1, Pfkbk1*	Fructose and mannose metabolism, Glucagon signaling pathway, AMPK signaling pathway (S. Zhang et al., 2022; Y. Zhang et al., 2024)
**alone**	*Grm1*	Long-term depression, Long-term potentiation (Yang et al., 2019, p. 20; L. Zhang et al., 2003),Gap junction, Taste transduction, Glutamatergic synapse (McDonald et al., 1998) , FoxO signaling pathway, Estrogen signaling pathway, Phospholipase D signaling pathway, Spinocerebellar ataxia, Retrograde endocannabinoid signaling
	Ndufs7	not statistically significant output
	Tnr	ECM-receptor interaction, Focal adhesion, MicroRNAs in cancer, PI3K-Akt signaling pathway (Ikeda et al., 2015), Human papillomavirus infection

Paper 2 Supplementary Materials

**Table S4 hCtx**		
**hCtx NJ**		
**Cluster**	**Genes**	**Function, signaling pathway, disease**
**1**	*Arhgap4, Cyp1a1, Cyp1a2, Cyp2a3, Ndufs7, Pfkfb1, Thbs2*	Retinol metabolism, Chemical carcinogenesis - reactive oxygen species (Lambert et al., 2023; Llido et al., 2023; Thomas et al., 2022; Wagner et al., 2021), Tryptophan metabolism, Metabolism of xenobiotics by cytochrome P450 (Johnson et al., 2012), Chemical carcinogenesis - DNA adducts (Asad et al., 2002), Steroid hormone biosynthesis, Chemical carcinogenesis - receptor activation
**2**	*Col4a3, Grm1, Ntsr1, Ptn*	Calcium signaling pathway (*KEGG PATHWAY: Calcium Signaling Pathway - Homo Sapiens (Human)*, n.d.; Liang et al., 2017; Rauti et al., 2020; H.-S. Shi et al., 2019)
**3**	*Camlg, Casp6, Ndufb8, Slc39a12, Slit3*	No statistically significant output
**4**	*Cacna2d4, Hyal4*	Glycosaminoglycan degradation, Arrhythmogenic right ventricular cardiomyopathy, Cardiac muscle contraction, Hypertrophic cardiomyopathy, Dilated cardiomyopathy, Lysosome, Adrenergic signaling in cardiomyocytes, Oxytocin signaling pathway
**Alone**	*Tnr*	ECM-receptor interaction, Focal adhesion, MicroRNAs in cancer, PI3K-Akt signaling pathway (Ikeda et al., 2015), Human papillomavirus infection
	Bmp5	TGF-beta signaling pathway, Hippo signaling pathway, Cytokine-cytokine receptor interaction
**hCtx LP**		
**Cluster**	**Genes**	**Function, signaling pathway, disease**

**5**	*Arhgap4, Ndufb8, Ptn, Slc39a12, Slit3*	Parkinson disease, Alzheimer disease
**6**	*Col4a3, Cyp2a3, Pfkfb1, Tnr*	ECM-receptor interaction, Focal adhesion, PI3K-Akt signaling pathway (Ikeda et al., 2015), Human papillomavirus infection, Fructose and mannose metabolism
**7**	*Cacna2d4, Cyp1a2, Hyal4*	Glycosaminoglycan degradation, Linoleic acid metabolism, Tryptophan metabolism, Drug metabolism - cytochrome P450 (M. Zhang et al., 2024), Metabolism of xenobiotics by cytochrome P450 (Johnson et al., 2012), Arrhythmogenic right ventricular cardiomyopathy, Chemical carcinogenesis - DNA adducts (Asad et al., 2002), Steroid hormone biosynthesis, Cardiac muscle contraction, Retinol metabolism
**alone**	*Bmp5*	TGF-beta signaling pathway, Hippo signaling pathway, Cytokine-cytokine receptor interaction
	Camlg	No statistically significant output
	Casp6	Apoptosis (Grojean et al., 2000; Rodrigues et al., 2002), Lipid and atherosclerosis
	Cyp1a1	Tryptophan metabolism, Ovarian steroidogenesis, Metabolism of xenobiotics by cytochrome P450 (Johnson et al., 2012), Chemical carcinogenesis - DNA adducts (Asad et al., 2002), Steroid hormone biosynthesis, Retinol metabolism, Lipid and atherosclerosis, Chemical carcinogenesis - receptor activation, Chemical carcinogenesis - reactive oxygen species (Lambert et al., 2023; Llido et al., 2023; Thomas et al., 2022; Wagner et al., 2021),
	Grm1	Long-term depression, Long-term potentiation (Yang et al., 2019, p. 20; L. Zhang et al., 2003), Gap junction, Taste transduction, Glutamatergic synapse (McDonald et al., 1998), FoxO signaling pathway, Estrogen signaling pathway, Phospholipase D signaling pathway, Spinocerebellar ataxia, Retrograde endocannabinoid signaling
	Ndufs7	Retrograde endocannabinoid signaling, Oxidative phosphorylation (Hansen, 2000; Zetterström & Ernster, 1956),, Non-alcoholic fatty liver disease (Ramírez-Mejía et al., 2024; Tian et al., 2016), Diabetic cardiomyopathy, Chemical carcinogenesis - reactive oxygen species (Lambert et al., 2023; Llido et al., 2023; Thomas et al., 2022; Wagner et al., 2021), Thermogenesis, Parkinson disease, Prion disease, Huntington disease
	Ntsr1	Amyotrophic lateral sclerosis, Calcium signaling pathway (Liang et al., 2017; Rauti et al., 2020; H.-B. Shi et al., 2006), Neuroactive ligand-receptor interaction
	Thbs2	Malaria, ECM-receptor interaction, Phagosome, Focal adhesion, Cytoskeleton in muscle cells, PI3K-Akt signaling pathway (Ikeda et al., 2015), Human papillomavirus infection
**hCtx SP**		
**Cluster**	**Genes**	**Function, signaling pathway, disease**
**8**	*Cyp1a1, Cyp1a2, Ndufs7*	Chemical carcinogenesis - reactive oxygen species (Lambert et al., 2023; Llido et al., 2023; Thomas et al., 2022; Wagner et al., 2021), Tryptophan metabolism, Metabolism of xenobiotics by cytochrome P450 (Johnson et al., 2012), Chemical

		carcinogenesis - DNA adducts (Asad et al., 2002), Steroid hormone biosynthesis, Retinol metabolism, Chemical carcinogenesis - receptor activation
**9**	*Arhgap4, Camlg, Casp6, Grm1, Slc39a12, Thbs2*	No statistically significant output
**10**	*Bmp5, Col4a3, Ndufb8, Tnr*	ECM-receptor interaction, Focal adhesion, PI3K-Akt signaling pathway (Ikeda et al., 2015), Human papillomavirus infection
**11**	*Cacna2d4, Cyp2a3, Hyal4, Pfkfb1, Ptn, Slit3*	No statistically significant output
**alone**	*Ntsr1*	Calcium signaling pathway (Liang et al., 2017; Rauti et al., 2020; H.-B. Shi et al., 2006), Neuroactive ligand-receptor interaction

Cll – cerebellum; *Arhgap4* – Rho-GTPase activating protein 4; *Bmp5*— bone morphogenetic protein 5; *Cacna2d4*—calcium voltage-dependent calcium channel complex alpha-2/delta subunit family; *Camlg*— calcium modulating ligand; *Casp6*—caspase 6; *Col4a3*—collagenase 4a3; *Cyp1a1/1a2/2a3/2a5* —cytochrome P450 1a1/1a2/2a3/2a5 (*Cyp2a3* in rats = *Cyp2a5* in humans); *Grm1*—glutamate metabotropic receptor 1; *Hyal4*—hyaluronic acid 4; *Ndufs7/b8* —NADH—ubiquinone oxidoreductase (complex I) subunit 7/8; *Ntsr1*—neurotensin receptor 1; *Pfkfb1*—6-phosphofructo-2-kinase/fructose- 2,6-biphosphatase 1; *Ptn*—pleiotrophin; *Slc39a12*—solute carrier family 39 member 12; *Slit3*—slit guidance ligand 3; *Thbs2*—thrombospondin 2; *Tnr*—tenascin R. Red text: reported in the literature of bilirubin; blue text: "gain of function" in LP vs. NJ; Green text: "gain of function" in SP vs. both NJ and LP.

References

Asad, S. F., Singh, S., Ahmad, A., & Hadi, S. M. (2002). Bilirubin/biliverdin–Cu(II) induced DNA breakage; reaction mechanism and biological significance. *Toxicology Letters*, *131*(3), 181–189. https://doi.org/10.1016/S0378-4274(02)00031-0

Grojean, S., Koziel, V., Vert, P., & Daval, J.-L. (2000). Bilirubin Induces Apoptosis via Activation of NMDA Receptors in Developing Rat Brain Neurons. *Experimental Neurology*, *166*(2), 334–341. https://doi.org/10.1006/exnr.2000.7518

Hansen, T. W. R. (2000). Bilirubin Oxidation in Brain. *Molecular Genetics and Metabolism*, *71*(1), 411–417. https://doi.org/10.1006/mgme.2000.3028

Ikeda, Y., Hamano, H., Satoh, A., Horinouchi, Y., Izawa-Ishizawa, Y., Kihira, Y., Ishizawa, K., Aihara, K., Tsuchiya, K., & Tamaki, T. (2015). Bilirubin exerts pro-angiogenic property through Akt-eNOS-dependent pathway. *Hypertension Research*, *38*(11), 733–740. https://doi.org/10.1038/hr.2015.74

Johnson, C. H., Patterson, A. D., Idle, J. R., & Gonzalez, F. J. (2012). Xenobiotic Metabolomics: Major Impact on the Metabolome. *Annual Review of Pharmacology and Toxicology*, *52*(Volume 52, 2012), 37–56. https://doi.org/10.1146/annurev-pharmtox-010611-134748

KEGG PATHWAY: Calcium signaling pathway—Homo sapiens (human). (n.d.). Retrieved 29 April 2025, from https://www.kegg.jp/pathway/hsa04020

Lambert, B., Semmler, A., Beer, C., & Voisey, J. (2023). Pyrroles as a Potential Biomarker for Oxidative Stress Disorders. *International Journal of Molecular Sciences*, *24*(3), Article 3. https://doi.org/10.3390/ijms24032712

Liang, M., Yin, X.-L., Shi, H.-B., Li, C.-Y., Li, X.-Y., Song, N.-Y., Shi, H.-S., Zhao, Y., Wang, L.-Y., & Yin, S.-K. (2017). Bilirubin augments Ca2+ load of developing bushy neurons by targeting specific subtype of voltage-gated calcium channels. *Scientific Reports*, *7*(1), 431. https://doi.org/10.1038/s41598-017-00275-9

Llido, J. P., Jayanti, S., Tiribelli, C., & Gazzin, S. (2023). Bilirubin and Redox Stress in Age-Related Brain Diseases. *Antioxidants*, *12*(8), Article 8. https://doi.org/10.3390/antiox12081525

McDonald, J. W., Shapiro, S. M., Silverstein, F. S., & Johnston, M. V. (1998). Role of Glutamate Receptor-Mediated Excitotoxicity in Bilirubin-Induced Brain Injury in the Gunn Rat Model. *Experimental Neurology*, *150*(1), 21–29. https://doi.org/10.1006/exnr.1997.6762

Ramírez-Mejía, M. M., Castillo-Castañeda, S. M., Pal, S. C., Qi, X., & Méndez-Sánchez, N. (2024). The Multifaceted Role of Bilirubin in Liver Disease: A Literature Review. *Journal of Clinical and Translational Hepatology*, *12*(11), 939–948. https://doi.org/10.14218/JCTH.2024.00156

Rauti, R., Qaisiya, M., Tiribelli, C., Ballerini, L., & Bellarosa, C. (2020). Bilirubin disrupts calcium homeostasis in neonatal hippocampal neurons: A new pathway of neurotoxicity. *Archives of Toxicology*, *94*(3), 845–855. https://doi.org/10.1007/s00204-020-02659-9

Rodrigues, C. M. P., Solá, S., & Brites, D. (2002). Bilirubin induces apoptosis via the mitochondrial pathway in developing rat brain neurons. *Hepatology*, *35*(5), 1186. https://doi.org/10.1053/jhep.2002.32967

Shi, H.-B., Kakazu, Y., Shibata, S., Matsumoto, N., Nakagawa, T., & Komune, S. (2006). Bilirubin potentiates inhibitory synaptic transmission in lateral superior olive neurons of the rat. *Neuroscience Research*, *55*(2), 161–170. https://doi.org/10.1016/j.neures.2006.02.015

Shi, H.-S., Lai, K., Yin, X.-L., Liang, M., Ye, H.-B., Shi, H.-B., Wang, L.-Y., & Yin, S.-K. (2019). Ca2+-dependent recruitment of voltage-gated sodium channels underlies bilirubin-induced overexcitation and neurotoxicity. *Cell Death & Disease*, *10*(10), 1–15. https://doi.org/10.1038/s41419-019-1979-1

Thomas, D. T., DelCimmuto, N. R., Flack, K. D., Stec, D. E., & Hinds, T. D. (2022). Reactive Oxygen Species (ROS) and Antioxidants as Immunomodulators in Exercise: Implications for Heme Oxygenase and Bilirubin. *Antioxidants*, *11*(2), Article 2. https://doi.org/10.3390/antiox11020179

Tian, J., Zhong, R., Liu, C., Tang, Y., Gong, J., Chang, J., Lou, J., Ke, J., Li, J., Zhang, Y., Yang, Y., Zhu, Y., Gong, Y., Xu, Y., Liu, P., Yu, X., Xiao, L., Du, M., Yang, L., … Miao, X. (2016). Association between bilirubin and risk of Non-Alcoholic Fatty Liver Disease based on a prospective cohort study. *Scientific Reports*, *6*(1), 31006. https://doi.org/10.1038/srep31006

Wagner, K.-H., Seyed Khoei, N., Hana, C. A., Doberer, D., Marculescu, R., Bulmer, A. C., Hörmann-Wallner, M., & Mölzer, C. (2021). Oxidative Stress and Related Biomarkers in Gilbert's Syndrome: A Secondary Analysis of Two Case-Control Studies. *Antioxidants*, *10*(9), Article 9. https://doi.org/10.3390/antiox10091474

Yang, L., Wu, D., Wang, B., Bu, X., Zhu, J., & Tang, J. (2019). The effects of hyperbilirubinaemia on synaptic plasticity in the dentate gyrus region of the rat hippocampus in vivo. *Archives of Medical Science*, *16*(1), 200–204. https://doi.org/10.5114/aoms.2019.88625

Zetterström, R., & Ernster, L. (1956). Bilirubin, an Uncoupler of Oxidative Phosphorylation in Isolated Mitochondria. *Nature*, *178*(4546), 1335–1337. https://doi.org/10.1038/1781335a0

Zhang, L., Liu, W., Tanswell, A. K., & Luo, X. (2003). The Effects of Bilirubin on Evoked Potentials and Long-Term Potentiation in Rat Hippocampus In Vivo. *Pediatric Research*, *53*(6), 939–944. https://doi.org/10.1203/01.PDR.0000061563.63230.86

Zhang, M., Rottschäfer ,Vivi, & and C. M. de Lange, E. (2024). The potential impact of CYP and UGT drug-metabolizing enzymes on brain target site drug exposure. *Drug Metabolism Reviews*, *56*(1), 1–30. https://doi.org/10.1080/03602532.2023.2297154

Zhang, S., Fan, Y., Zheng, B., Wang, Y., Miao, C., Su, Y., Li, K., E., Y., Wang, X., He, X., Wu, X., Xu, C., Tang, Y., Liu, W.-T., Kong, X., & Hu, L. (2022). Bilirubin Improves Gap Junction to Alleviate Doxorubicin-Induced Cardiotoxicity by Regulating AMPK-Axl-SOCS3-Cx43 Axis. *Frontiers in Pharmacology*, *13*. https://doi.org/10.3389/fphar.2022.828890

Zhang, Y., Chen, Y., Chen, X., Gao, Y., Luo, J., Lu, S., Li, Q., Li, P., Bai, M., Jiang, T., Zhang, N., Zhang, B., Chen, B., Zhou, H., Jiang, H., & Lin, N. (2024). Unconjugated bilirubin promotes uric acid restoration by activating hepatic AMPK pathway. *Free Radical Biology and Medicine*, *224*, 644–659. https://doi.org/10.1016/j.freeradbiomed.2024.09.023
